# A voyage of reprogrammable metabolic bioengineering reshapes plant defense: from editing tools to synthetic systems

**DOI:** 10.3389/fpls.2026.1842722

**Published:** 2026-08-26

**Authors:** Pandiyan Raghuraman, SeonJoo Park

**Affiliations:** Department of Life Sciences, Yeungnam University, Gyeongsan, Gyeongsangbuk-do, Republic of Korea

**Keywords:** metabolic bioengineering, plant immunity, spatial metabolomics, synthetic gene circuits, volatile-mediated defense

## Abstract

Metabolic bioengineering has emerged as a transformative approach for reshaping plant defense by targeting intrinsic biosynthetic pathways to enhance immunity in modern agriculture. Moving beyond proof-of-concept metabolomics to broad-spectrum programmable pathway engineering addresses gaps in plant rational design and optimizes resilience in response to diverse environmental cues. This review aims to comprehensively highlight the transition of innovative approaches to phenolics, alkaloids, flavonoids, terpenoids, and benzoxazinoids, inferring adaptive reprogramming that mediates the growth-defense balance and functions as molecular sentinels in plants. Furthermore, decoding the volatile metabolome reveals a dynamic signaling interface that influences defense responses and stress-induced plant-microbe interactions, with the shikimate, jasmonate, and salicylate pathways functioning as central hubs for microbial deterrence and priming immune memory. Recent developments in multi-scalar genome-editing strategies, including CRISPR-driven combinatorial edits, enzyme orthogonalization, fluxomics, and spatially resolved multi-omics, reconfigure central and specialized metabolic fluxes toward improved defense function and regulation. Additionally, emerging tools, such as WUSCHEL2 and BABY BOOM transcriptional modules, and artificial engineering strategies integrating deep learning model-driven predictions facilitate rapid development of synthetic genetic circuits and support a predictive engineering of plants. Moreover, Mass spectrometry imaging (MSI) in spatial metabolomics enables to obtain structures and locations of unidentified endogenous metabolites within cells and tissues. Overall, this review emphasizes a diverse array of primary and secondary metabolites, spanning molecular concepts to recent advances in plant immune mechanisms. It also illustrates new frontiers in programmable metabolic engineering that accelerate the understanding of plant-microbe-metabolite cross-talks, offering strategies to improve plant resistance and advance sustainable agricultural solutions.

## Introduction

Plants, as sessile multicellular organisms, are continuously exposed to adverse environmental conditions. Consequently, they have evolved a metabolic responsiveness that coordinate a complex network of defense responses to external stimuli; including innate immunity against biotic threats (pathogens), the molecular exchange with plant associated microbiota, and the biosynthesis of specialized metabolites. The production of specialized metabolites encompassing diverse classes including, cyanogenic glycosides, terpenoids and alkaloids, glucosinolates, and flavonoids confers ecological protection against potential biotic and abiotic stressors (pathogens and herbivorous insects). Beyond ecological defense, many of these secondary metabolites exhibit diverse pharmacologic and industrial applications (Mithöfer and Boland, 2012; Pascale et al., 2020). The potential to engineer metabolic pathways through rational modification of endogenous biosynthetic processes using metabolic bio-engineering (MBE) is substantial. In this review, metabolic bioengineering is considered an expanded conceptual framework that integrates classical metabolic engineering with synthetic biology, genome editing, systems biology, multi-omics technologies, computational modeling, and microbiome-based approaches to enable predictive and programmable regulation of plant metabolic networks. However, the limited capacity to modify plant defense phenotypes reflects the inherent complexity of plant metabolic pathways, including subcellular compartmentalization, genetic redundancy among enzymes involved in these pathways, and regulatory crosstalk between developmental and responsive programs (Jirschitzka et al., 2013; Selma et al., 2023). ME originated as an applied discipline in biochemical genetics and radioactive tracers in the mid-20th century; however, the first practical tools used for ME emerged in the 1980’s enabling plant transformation, promoter dissection, and cloning platform developments (DellaPenna, 2001). Early pathway studies, including, fatty acid and carotenoid biosynthesis served as experimental systems to evaluate heterologous gene expression and functional reconstruction of pathways in microbial systems and mutant libraries (Browse and Somerville, 1991; Chamovitz et al., 1991). Integrated analysis of KNApSAcK-KAMPO-JAMU databases (DBs) suggests that plants produce approximately 200 000 to one million compounds; while several thousand of these compounds have been identified, only a small fraction have been characterized for their physiological roles or biological activities (Saito and Matsuda, 2010; Afendi et al., 2012).

Although significant progress has been made in traditional breeding methods, chemical crop protection, and marker-assisted selection, these techniques still have several limitations. Breeding cycles can take a long time and require substantial resources. The process also depends on the amount of genetic diversity. Additionally, when a pathogen evolves quickly enough, it can develop resistance to a targeted gene bred into crops. Pesticides provide an efficient means of protecting crops from pathogens. However, they pose serious threats to the environment. These include soil and water contamination, harm to wildlife and humans, the development of resistant strains, and unsustainable use. Marker-assisted selection has helped researchers identify resistance-related genes and regions. Still, this method typically focuses on a single characteristic or mechanism. It does not provide a complete understanding of the complex relationships in plant defenses (Collard and Mackill, 2008). Research continues to demonstrate that plant resistance is influenced by various factors. These include host metabolic processes, microbial communities in the soil/plant interface, and environmental conditions. Therefore, more holistic and dynamic approaches to protecting crops are needed. Rhizosphere-associated microbial communities play a significant role in plant health and nutrition. Metagenomics research demonstrates its ability to suppress diseases (Roman-Reyna and Crandall, 2024). These results highlight the importance of plant-microbe-mediated resistance mechanisms in agriculture. Furthermore, the discovery of volatile-based biocontrol mechanisms in beneficial microorganisms, such as those in the genus Bacillus, supports the use of microbial metabolites. This approach can enhance plant defenses beyond typical disease management practices (Liu et al., 2025b).

Recent approaches in plant metabolic bioengineering are categorized into four conceptual areas: (i) transcriptional circuitry engineering, (ii) modular assembly of biochemical pathways, (iii) sub-cellular localization and tissue-specific targeting of metabolites, and (iv) interaction with hormonal stress and signaling networks (Zhu et al., 2015; Arendt et al., 2016; Selma et al., 2023). Transcriptional regulation is often mediated using the transcription factors (TFs), including MYB, WRKY, bHLH, and AP2/ERF, enabling coordinated expression of multiple steps within biochemical pathways. For example, the transcriptional regulators CrMYC2, ORCA3, and BIS1/BIS2 regulate terpenoid indole alkaloid biosynthesis in *Catharanthus roseus*, while, AaERF1/2 regulate artemisinin biosynthesis in *Artemisia annua* through jasmonate-sensitive cis-elements (Zhang et al., 2011; Yu et al., 2012; Jirschitzka et al., 2013). Introducing heterologous or synthetic TFs into a chassis plant genome, capable of functioning independently of native regulatory elements, is essential for autonomous regulation of metabolite synthesis (Liu et al., 2023b). This strategy is particularly important for development of defense mechanisms, as transcriptional activation is often tightly coupled to stress perception, which can be transient. The second axis, modular multigene reconstruction, addresses the challenge of expressing sequential enzymatic steps in linear or branched metabolic pathways. Synthetic biological frameworks have enabled the successful construction of pathways such as the noscapine biosynthetic gene cluster (BGC) in *Papaver somniferum* and the artemisinin biosynthetic pathway in *Nicotiana benthamiana* using Golden Gate, Loop Assembly, and polycistronic tRNA-spaced systems (Winzer et al., 2012; Liu et al., 2025a). In addition to enabling dynamic flux balancing through tunable promoters, translational fusions, and metabolon-forming linkers to circumvent pathway bottlenecks and metabolic burden, a third objective of ME is cell-type and organelle specific localization of biosynthesis. Reactive and auto toxic intermediate produced during ME often require localized regulation to prevent systemic toxicity as observed in glandular trichome-localized terpene production and vacuolar alkaloid sequestration. Such spatial localization can be achieved using cell type specific promoters (NtLTP1, Pdf2.1), signal peptide sequences for plastid or ER targeting and engineered protein scaffolds that co-localizes enzymes to maximize flux and minimize catabolite diffusion (Choi et al., 2012; Kang et al., 2019). Selma et al, further emphasize that successful ME designs increasingly exploit intrinsic cellular logistical systems – membrane transporters, sub-organelle micro-domains, and vesicular trafficking – to direct substrate flow and reduce metabolic interference. The fourth dimension of ME involves integrating biosynthetic modules into broader immune and hormonal signaling networks, specifically those regulated by jasmonic acid (JA), salicylic acid (SA), and ethylene (ET). Phytohormone responsive TF, including, MYC2, WRKY70, and ERF1 mediate crosstalk between PAMP-triggered immunity and the metabolic output of these pathways, positioning phytohormone regulatory pathways as tunable switches for activating specialized metabolism (Franco-Zorrilla et al., 2014; Aerts et al., 2021). An emerging paradigm in plant metabolic bioengineering emphasizes that successful defense engineering depends not only on increasing metabolite abundance but also on controlling where, when, and in what biochemical form defense metabolites are synthesized, transported, conjugated, stored, secreted, volatilized, or released. Such spatiotemporal regulation enables plants to deploy metabolites precisely at infection sites while minimizing constitutive metabolic expenditure and unintended effects on growth and development. Consequently, engineering pathogen-inducible promoters, tissue-specific expression systems, subcellular compartmentalization, vesicle-mediated trafficking, transporter-mediated metabolite allocation, and controlled metabolite deployment offers a more sustainable strategy for optimizing growth–defense balance than constitutive pathway overexpression alone. This systems-oriented perspective provides a unifying framework for programmable metabolic bioengineering and underpins many of the advanced engineering approaches discussed throughout this review. Furthermore, immune signaling cascades that activate defense-specific transcriptional reprogramming through MAPKs, CDPKs, and reactive oxygen species (ROS), generate diverse defense metabolites and antimicrobial compounds, including camalexin, scopoletin, and glucosinolates (Großkinsky et al., 2012; Boudsocq and Sheen, 2013; Manna et al., 2023). Also, protein engineering strategies are applied to these nodes, for example, modifying the allosteric sites of dihydrodipicolinate synthase or ADPG pyrophosphorylase to increase flux while bypassing negative feedback (Brinch-Pedersen et al., 1996; Dong and Ronald, 2019; Abdul Aziz et al., 2022). Future ME is poised to integrate high resolution spatial-omics, machine learning (ML)-based metabolic modeling, and single cell transcriptomics to enhance predictive design and integrative control of plant metabolic pathways. Incorporating these technologies with CRISPR-driven multiplexed genome editing, synthetic TF libraries, and feedback regulated biosensors, is transforming ME from static gene stacking to programmable cellular logic — a paradigm capable of redefining the boundaries of plant defense and advancing sustainable agriculture. While CRISPR/Cas-based genome editing has become a dominant platform for targeted manipulation of defense-associated pathways, numerous reviews have already comprehensively covered its applications in crop improvement. Despite substantial advances in metabolic engineering, a fundamental challenge remains: how can metabolic fluxes and specialized metabolite deployment be rationally programmed to enhance plant immunity while minimizing growth–defense trade-offs and maintaining agronomic performance under diverse environmental conditions? This review addresses this central question by critically examining the convergence of metabolic bioengineering, synthetic biology, genome editing, systems biology, multi-omics technologies, computational modeling, and microbiome engineering as complementary strategies for programmable plant defense. Rather than presenting these technologies independently, we evaluate their experimental maturity, translational potential, and current limitations, highlighting validated applications, emerging proof-of-concept studies, and future opportunities for sustainable crop protection. The review is therefore organized to progress from defense-associated metabolic pathways and regulatory networks to programmable engineering strategies, systems-level analytical platforms, translational challenges, and future research priorities, providing an integrated framework for next-generation metabolic bioengineering.

In this updated review we consolidate and critically assess the literatures from the last decade progress to recent advances in metabolic bioengineering identified from comprehensive searches of major scientific databases (pubag, EnsemblPlants etc), including Web of Science, Scopus, PubMed, and Google Scholar. Priority was given to peer-reviewed articles, with emphasis that integrates plant metabolic bioengineering, synthetic biology, genome editing, multi-omics technologies, computational modeling, and plant–microbe interactions. Earlier seminal studies were also included where necessary to provide historical context and highlight the evolution of key concepts. The selected literature was critically evaluated for scientific relevance, methodological quality, and contribution to the current understanding of programmable metabolic bioengineering for plant defense.

## Metabolic bioengineering in plant immunity: concepts and rationale

The plant defense system is a dynamic, multi-component communication network that facilitates the recognition and rapid conversion of pathogen-derived signals into localized cell death and antimicrobial compound production while also establishing long-distance immune memory through SAR (systemic acquired resistance) and ISR (induced systemic resistance) (Bolton, 2009). Beyond the biochemical nexus of metabolism and defense circuitry, a complex network of primary and secondary or specialized metabolites is employed during pathogen encounter to produce diverse antimicrobial compounds and enhance immune responses. Accordingly, ME emerges not merely as a strategy to increase metabolite production but as a sophisticated approach to remodel the metabolic-immune interface, thereby enhancing plant resilience (Zhu et al., 2020). Plants have co-evolved with environmental microorganisms by developing multiple layers of defense that include structural barriers (e.g., lignin, callose, cutin, and suberin), specialized defense metabolites (e.g., phenylpropanoids, flavonoids, alkaloids, glucosinolates, benzoxazinoids, terpenoids, phytoalexins, and volatile organic compounds), defense-associated proteins (e.g., pathogenesis-related proteins, defensins, thionins, chitinases, and polygalacturonase-inhibiting proteins), elicitor molecules (e.g., chitosan and laminarin), and immune signaling molecules including salicylic acid (SA), jasmonic acid (JA), ethylene (ET), abscisic acid (ABA), and pipecolic acid/N-hydroxypipecolic acid (NHP), which collectively coordinate multilayered immune responses against diverse biotic stresses (Ahuja et al., 2012; Maeda and Dudareva, 2012). Through ME, targeted manipulation of biosynthetic nodes — comprising rate-limiting enzymes, transcriptional activators and hormone-responsive switches can elevate these metabolite levels above basal thresholds, enhancing disease resistance (Fan, 2024; Ramakrishnan et al., 2025). Approaches such as overexpression of endogenous genes, introduction of novel or orthogonal biosynthetic pathways, or CRISPR-mediated modifications to the regulatory hierarchies controlling metabolite production. To prevent cellular toxicity and maximize metabolic flux partitioning and consequently metabolite production, strategies include tissue-specific promoters, cellular engineering, metabolon-guided scaffolds, and enzyme re-localization to compartments that minimize interference with other metabolic processes (Lee et al., 2012; Jirschitzka et al., 2013; Siu et al., 2015; Venayak et al., 2015). The transcriptional control of these metabolic reprogramming events is often orchestrated by master regulators, such as NAC, MYB, or HSF, which integrate environmental cues into the regulation of metabolite biosynthesis. Engineering TFs or their corresponding cis-regulatory motifs enables coordinated expression of multiple genes within an entire BGC as demonstrated for alkaloid and terpenoid pathways (Nützmann et al., 2016).

Recent advances in genome-scale metabolic modeling, spatiotemporal transcriptomics, and metabolic flux analysis (fluxomics) provide researchers with predictive tools to determine key bottlenecks and optimize dynamic metabolic control through the application of QTL (Saito and Matsuda, 2010; Peace et al., 2014; Lv et al., 2020; Yang et al., 2020). Specifically, combining multiple omics datasets with flux balance models provides valuable insights into how context-specific fluxes can be redirected in immune-primed plants, allowing rational manipulation of defense-associated metabolite levels without disrupting core metabolic functions of the plant. The advent of genome editing is transforming synthetic ME in plants by providing researchers with unprecedented precision and modularity in manipulating genes and pathways. The development of new tools such as base and prime editors, as well as dCas9-based gene circuits, enables fine regulation of gene expression, epigenetic states, and pathway activation and repression without introducing double-strand DNA breaks (Tan et al., 2023). These tools also facilitate the modification of SNPs in regulatory regions of the genome, the reprogramming of the epigenetic state of metabolic loci, and the simultaneous activation or repression of multiple pathways using CRISPRa-based systems (e.g., dCasEV2.1 and CRISPR-Act3.0), thereby providing precise control over immune-mediated metabolic responses (Liu et al., 2025a). Similarly, programmable logic-based control is increasingly applied to plant gene circuits, enabling responses to environmental or developmental cues. Research on optogenetic regulators, hormone-inducible switches, and feedback-controlled biosensors provides mechanisms for stress-responsive immune gene expression, thereby reducing metabolic costs and mitigating potential unintended consequences of overactive immune responses (Andres et al., 2019). Apart from chemical defense, ME also encompasses to structural fortification. Enhancing monolignol biosynthesis or modifying callose deposition can increase plant physical resistance, limiting pathogen entry. The development of engineered plants with elevated levels of SA, JA and ET at specific times, provides additional systems level opportunities to enhance immunity (Creelman and Mullet, 1995; Menke et al., 1999; Meraj et al., 2020). Despite advances in understanding the complexities of plant metabolic networks, including their inherent polygenicity, compartmentalization and feedback sensitivity, ME now provides an opportunity to emulate plant modular logic using orthogonal synthetic tools to encode defense as a programmable, evolvable trait. This represents a major shift from reactive metabolite control to proactive immunity programming and establishing a new paradigm for crop protection amid climate instability, declining pesticide efficacy and the pursuit of sustainable agriculture (Abdelrahman et al., 2021; Huang et al., 2021).

## Defense-associated metabolites in plant-microbe interactions

Plants orchestrate immune response through large-scale, reorganization of metabolic pathways, coordinating microbial perception, systemic defenses, and the restructuring of microbial communities on the plant surface (holobionts) (Piasecka et al., 2015). Next-generation metabolites have been identified as key components in the plant metabolic architecture, directing cross-kingdom communication and receptor network connectivity, facilitating microbial consortia formation via pattern recognition receptors (PRRs), modulating the metabolites flux, and introducing resilience into the metabolic pathway networks (Teixeira et al., 2019). Advances in metabolomics have transformed the study of plant–microbe interactions from anecdotal reports of individual metabolites to systems-level biochemistry, where the metabolite exchange kinetics, thermodynamics of biochemical reactions, and metabolite allocation cost collectively determine the immune response outcomes (Tugizimana et al., 2018; Nehela et al., 2023; Zhu et al., 2023). A key conceptual insight is that immune receptors protect the function of metabolic enzymes, thus integrating pattern-triggered immunity (PTI) and effector triggered immunity (ETI) into the regulation of metabolic allocation. For example, the NLR PigmR protects the deubiquitinase PICI1, which preserves methionine synthase (METS1) function from effector mediated degradation, maintaining methionine flux into ET biosynthesis and conferring broad-spectrum resistance to the fungus *Magnaporthe oryzae* (Zhai et al., 2022).

This exemplifies a paradigm shift in understanding how immunity functions in organisms, it demonstrating that protection extends beyond signaling cascades to include stabilization of flux-critical enzymes in metabolic pathways. Such mechanism ensures that failure in a single metabolic pathway does not compromise systemic defense across the functional system. The metabolic dimension of these guarding mechanisms extends beyond ET biosynthesis. The stabilization of METS1 by PICI1 assure that methionine availability for S-adenosylmethionine (SAM) synthesis, which supports hormone production and methylation of nucleic acids and proteins. Therefore, immune stimulation is linked to DNA and histone methylation, and the establishment of long-term resistance memory as epigenetic mark (Escaray et al., 2022). Protections associated with amino acid metabolism further support to immunity: lysine catabolism generates SAR regulators, including pipecolic acid (Návarová et al., 2012), while methionine flux sustains ET biosynthesis. The disruption of these mechanisms impairs immune function, while strengthening them through either genetic overexpression or exogenous supplementation enhances the organismal capacity to withstand environmental perturbations. Together, these findings support for the methionine-SAM-ET axis as a model of immune-metabolic resilience (Broquist, 1991).

Specialized metabolic enzymes exemplify the convergence of defense and evolution. CYP79 mono-oxygenase channel amino acid precursors into cyanogenic glycosides and glucosinolates, while oxidosqualene cyclases (OSCs), and diterpene synthases (DTSs), generate scaffold for triterpenoids and diterpenoids, respectively. The chimeric STORR enzyme performs stereospecific transformations at intermediate stages of alkaloid biosynthesis (Bak et al., 1998; Zerbe and Bohlmann, 2015; Bharadwaj et al., 2021). However, novelty lies not only in the catalytic diversity of these enzymes, but also in the genomic organization of their clusters. These clusters encompass all enzymes required to extend the chemical repertoire of plant defense, including cytochrome P450s, glycosyltransferases, methyltransferases, acyltransferases, dioxygenases, reductases, and transaminases. Some clusters incorporate “moonlighting” enzymes recruited from unrelated pathways, while others contain duplicated genes that have evolved into sequential, specialized steps, such as the tandem arrays of CYP enzymes mediating benzoxazinoid biosynthesis in maize. Furthermore, modular design is evident in acylation cassettes, which contain adjacent genes encoding successive enzymatic reactions to append defensive groups to substrates. While strictly collinear gene arrangements are rare, their occurrence in alkaloid clusters suggests that gene order may be influence the timing and assembly of the pathway. Overall, tailoring, duplication, modularity, and moonlighting demonstrate how clusters represent evolutionary innovations that integrate chemical diversity with enhanced pathogens resistance. The rhizosphere represents a highly dynamic interface in this continuous biochemical interaction, where root exudates serve as semiochemicals and nutrient cues, coordinating quorum sensing, biofilm formation, and microbial community assembly (McLaughlin et al., 2023). Flavonoids embody multifunctionality, they activate the NodD-mediated symbiosis in legumes, modulate auxin signaling and protect plants against UV stress. Additionally, they disrupt pathogen membrane structure integrity through chelation and redox toxicity (Hassan and Mathesius, 2012). Benzoxazinoids (BXs) and phenylpropanoids can modify microbial community composition and reinforce plant cell walls, however, their biosynthesis requires a diversion of photosynthetic resources and increased respiration, imposing carbon cost (Rojas et al., 2014). Therefore, the chemical composition of exudates reflects a cost-benefit trade-off for the host, shaping microbial consortia to enhance systemic plant defense. Recent analytical advances enable the detection of these shifts in resource allocation. UHPLC-HRMS, NMR, and isotopic labelling allow identification of low abundance conjugates formed during microbial transformation and alterations in biochemical pathways (Marti et al., 2013; Šimura et al., 2018; Allwood et al., 2021). These multi-omics approaches enhanced the ability to infer causal relationships between metabolite accumulation and resistance phenotypes (Cuperlovic-Culf et al., 2016). At the systemic level, SA jasmonate antagonism determines the type of immune response that occurs in plants, such as biotrophic or necrotrophic defense (Huot et al., 2014; Xu et al., 2022b).

The use of constraint-based and hybrid modeling frameworks builds on this knowledge to create predictive systems biology models that account for enzyme crowding, thermodynamic flux consistency, and protein allocating costs (Blonde et al., 2025). Newer-generation models, including GECKO, ETFL, and Resource Allocation Models (RAMs), explicitly simulate the energetic and regulatory costs associated with protein synthesis, effector interference and enzymes protection. These models simulate dynamic trade-offs between pathogen strategies and plant metabolism. Unlike classical genome-scale metabolic models (GEMs), which represent only optimal-steady state fluxes, they reveal how pathogen pressures alter metabolic fluxes. Incorporating NLR safeguards in modeling, allows immunity to be represented as the maintenance of continuous flux in the presence of antagonist effectors. For example, the PigmR–PICI1–METS1 safeguard is serve as quantifiable stabilizer of the methionine-ET flux (Zhai et al., 2022). Furthermore, spatial heterogeneity is the use of isotope-enabled nanoscale metabolomics, imaging mass spectrometry and single cell transcriptomics to reveal system-level dynamics. Consequently, plant microbe interactions can be conceptualized as real-time constrained optimization problems, with metabolic responses regulated by both nutritional and immune signals. However, activation of immunity introduces additional energetic costs on top of those associated with nutrient acquisition. A major part of this cost arises from diverting a substantial amounts of carbon and nitrogen from growth processes to produce essential metabolites (Nunes-Nesi et al., 2010). This diversion illustrates how lysine and methionine metabolism converges on the synthesis of immune signal molecules and hormones, thereby linking metabolic homeostasis to systemic responses. Therefore, immune resilience depends not only on the presence of specific metabolites, but also on the stability of enzymes catalysing their synthesis, the ability to maintain safe levels of metabolite flux through mechanisms such as flux control coefficients, and cellular allocation plasticity. These concepts have been applied to ME using a continuum of discovery-to-deployment. Therefore, identification of causal metabolites and transporters via metabolomic analyses enables the generation of desired phenotypes through breeding or selection. Concurrently, genome editing technologies (CRISPR and prime editing) allow redirection of flux toward the biosynthesis of protective compounds such as phenylpropanoids, terpenoids, phytoalexins and phenolamides (Selma et al., 2023; Li et al., 2024). Agronomically relevant traits, which are primarily reflect allocation patterns, can therefore be optimised for both abiotic stress tolerance and desirable quality characteristics (Farré et al., 2015). Deployment of these new traits requires chromatin-level editing and high-resolution spatial metabolite imaging to link local metabolic rewiring with the desired systemic outcomes (Castro-Moretti et al., 2020).

Plant–microbe interaction represents constrained optimization problem integrating the metabolic networks of both organisms. The host employs multiple mechanisms to limit photosynthetic production, increase glycolytic and tricarboxylic acid (TCA) cycle flux, redirect aromatic amino acids and acetyl-CoA toward defensive specialized metabolites, and modulate transporter activity to bias the composition of its microbial community. Conversely, pathogens have evolved diverse strategies to deploy effector proteins that disrupt the ability of the host to allocate resources effectively (Hartline et al., 2021). Thus, the outcome of the interaction depends on the extent to which critical metabolic nodes are protected by NLR proteins. When sufficient protection present, flux through the shikimate pathway increases, flavonoids biosynthesis is enhanced, and hormonal balance shifts to strengthen the capacity of the host to contain the pathogen. In the absence of sufficient NLR-mediated protection, these biochemical reprogramming events do not occur (Shi et al., 2023). Collectively, these findings support a three- tiered strategy for engineering crop resistance: (i) deploy flux-aware metabolomics to determine which biochemical pathways involved in host-pathogen interactions; (ii) evaluate the effectiveness of potential interventions using *in silico/simulation* analysis that incorporate constraint-based models to analyse gene expression and conformational dynamics; and (iii) implement genome editing and classical breeding approaches to reconfigure host metabolism, thus directing limited resources toward survival or pathogen defense (Escaray et al., 2022; Zhai et al., 2022; Blonde et al., 2025).

## Context dependent metabolic modulation toward pathogen resistance

ME for plant defense has advanced the understanding of regulatory mechanism and empowered the bio-design of target-oriented defense pathways and protective compounds to enhance resistance and crop productivity. The following sections provide a detailed overview of engineering strategies employed to date to increase plant resistance against pathogens.

## Defense metabolites in plant resistance to pathogen stresses

Plants deploy an intricate array of secondary metabolites that extend far beyond basal metabolism, functioning as dynamic modulators of immune competence under both biotic and abiotic stress. Among these, specific proteins and polysaccharides exhibit pronounced antiviral, antibacterial, and antifungal activities, mediating resistance through tightly coordinated signaling cascades (Pospieszny et al., 1991; van Loon et al., 2006; Wang et al., 2012; Yuan et al., 2022). Defense-related proteins (DRPs), comprising 17 distinct families, act as frontline molecular sentinels rapidly induced by SA, hydrogen peroxide, or viral RNA-associated signals. Representative examples include beetin-27 in *Beta vulgaris*, which confers broad-spectrum resistance, and CAP-34 and CT-VIA-62-type DRPs from *Clerodendrum aculeatum* and *Medicago truncatula*, respectively. These proteins exhibit lectin-like affinity for mannose residues and potent antiviral activity (Prasad et al., 2014). Similarly, DRPs isolated from *Celosia cristata* and *Phytolacca americana* inhibit replication of diverse plant and mammalian viruses, underscoring their structural conservation and mechanistic versatility (Balasubrahmanyam et al., 2000; Mansouri et al., 2012). In parallel, carbohydrate-derived polysaccharides constitute an additional vital layer of metabolic defense. Their low toxicity, broad spectrum activity, and functional pleiotropy—including antioxidative, immunostimulatory, and antiviral capacities—render them integral components of the plant biochemical arsenal (Shen et al., 2024). Particularly, polysaccharides extracted from *Lycium barbarum*, *Caesalpinia ferrea, Astragalus propinquus*, and *Arctium lappa* mitigate tobacco mosaic virus (TMV) and bursal disease virus infection by transcriptionally upregulating DRP and pathogenesis-related enzyme expression (Huang et al., 2008; Wang et al., 2009). In fungal defense, constitutive expression of defensin-type PR proteins (PR-12, PR-13, PR-14) and chitinases (PR-3) reinforces the cell wall and generates localized hydrogen peroxide bursts that deter pathogens, including *Alternaria brassicicola, Verticillium dahliae*, and *Rhizoctonia solani* (Penninckx et al., 1996; Wang et al., 1999; Gao et al., 2000). Beyond antiviral and antifungal resistance, metabolic reprogramming involving amino acid fluxes, ROS signaling, and PR-protein induction collectively sustains immunity against Pseudomonas syringae and other pathogens (Ausubel, 2005; Jones and Dangl, 2006). Collectively, such metabolite-driven immune orchestration exemplifies the context-dependent plasticity of primary metabolism in fortifying plants against multifaceted pathogenic biotic and abiotic stressors.

While these defense responses operate through interconnected molecular networks, the associated defense components differ substantially in their biochemical nature and functional roles. Defense proteins, including pathogenesis-related (PR) proteins, defensins, thionins, chitinases, ribonuclease-like proteins, lipid-transfer proteins, and polygalacturonase-inhibiting proteins, primarily function as antimicrobial effectors or immune modulators, whereas structural polymers such as callose, lignin, cutin, and suberin reinforce physical barriers that restrict pathogen invasion. In contrast, specialized metabolites including phenylpropanoids, flavonoids, alkaloids, glucosinolates, benzoxazinoids, terpenoids, phytoalexins, and volatile organic compounds (VOCs) constitute the principal biochemical outputs of defense-associated metabolic reprogramming. Their biosynthesis and deployment are dynamically coordinated by immune signaling molecules such as salicylic acid (SA), jasmonic acid (JA), ethylene (ET), abscisic acid (ABA), and pipecolic acid/N-hydroxypipecolic acid (NHP), together with defense elicitors including chitosan, laminarin, and alginate, thereby enabling spatially and temporally regulated immune responses against diverse pathogens.

Building upon the metabolic and signaling frameworks discussed in the preceding sections, specialized metabolites represent the principal biochemical outputs of defense-associated metabolic reprogramming. Rather than reiterating their biosynthetic origins, this section focuses on their direct functional contributions to pathogen suppression, immune amplification, and resistance establishment. These metabolites operate as dynamic defense effectors whose accumulation is regulated by immune signaling networks, metabolic flux allocation, and environmental cues. These structurally diverse compounds—including phenylpropanoids, alkaloids, flavonoids, terpenoids, saponins, and phytoalexins—constitute a metabolically flexible interface between plant signaling and microbial suppression (Hartmann, 2008). Their biosynthesis is frequently activated through microbe-associated molecular pattern (MAMP) perception or effector-triggered immunity (ETI), converging with SA, jasmonate, and ET signaling pathways to establish localized and systemic protection (Humphry et al., 2010). Phytoalexins, synthesized *de novo* upon infection, and pre-formed phytoanticipins collectively fortify tissues by disrupting pathogen membranes, impairing enzymatic redox processes, and inducing oxidative bursts that restrict colonization (Ahuja et al., 2012). For instance, phenolics and flavonoids can chelate metal ions, destabilize microbial cell walls, and form covalent adducts with sulfhydryl or amino groups of pathogen proteins, impairing catalytic fidelity and replication. Alkaloids including 7-deoxy-trans-dihydronarciclasine and bruceine-D exhibit potent antiviral activity against TMV, PVY, and CMV, while terpenoid phytoalexins such as zealexins in maize confer resistance to *Fusarium graminearum* and *Aspergillus flavus* through cell-wall reinforcement and ROS-mediated signaling (Pierpoint, 2000; Crozier et al., 2006). Similarly, saponins including α-tomatine and avenacins, perturb fungal membranes via G-protein-coupled cascades resulting in cytoplasmic leakage and calcium influx (Osbourn, 1996; Ito et al., 2007).

Beyond their role as phytoalexins, stilbenoids are an emerging model for integrating specialised metabolism, gene regulation, and metabolic engineering. Multi-omics work in grapevine shows that stilbene biosynthesis relies on regulatory networks of R2R3-MYB and WRKY transcription factors, cis-regulatory elements, and gene co-expression modules that guide the phenylpropanoid pathway. Genome-wide studies confirm that MYB14, MYB15, and MYB13 directly control stilbene synthase (STS) and phenylpropanoid pathway genes, and that these genes are central to stilbene accumulation. Co-expression and regulatory analyses reveal WRKY-mediated modules that fine-tune resveratrol biosynthesis in response to stress (Wong et al., 2016, Wong et al., 2017; Vannozzi et al., 2018; Orduña et al., 2022). Recent DAP-seq, transcriptomics, and gene co-expression analyses show Subgroup 2 R2R3-MYBs directly regulate genes in the shikimate pathway, early phenylpropanoid metabolism, and stilbenoid biosynthesis, linking primary metabolic flux to specialised defense metabolite production (Orduña et al., 2022). Aggregated co-expression network analysis now offers predictive frameworks for identifying regulatory landscapes and engineering targets in non-model crops (Orduña et al., 2023). Together, these studies show that multi-omics network reconstruction can identify key engineering targets to enhance defense-associated phenylpropanoid and stilbenoid metabolism. Collectively, these findings position specialized metabolites not merely as chemical end products but as dynamically inducible molecular sentinels that link innate immune perception with sustained pathogen suppression, reflecting the remarkable biochemical plasticity of plant immune metabolism.

## Integrative metabolomics insights into plant–pathogen reprogramming

The previous section discussed the biological roles of defense-associated metabolites in plant–microbe interactions. This section describes how advanced metabolomics and multi-omics approaches quantify these metabolic changes and identify regulatory nodes linked to disease resistance. High-resolution omics technologies have transformed the understanding of how pathogens remodel host metabolism to suppress immunity or enhance resistance. In maize infected with Sugarcane mosaic virus (SCMV), transcriptomic analyses reveal pronounced repression of photosynthetic genes corresponding to chlorotic lesions, while ribosome profiling uncovers a compensatory translational upregulation of phenylpropanoid enzymes, including phenylalanine ammonia-lyase (PAL) and 4-coumarate: CoA ligase (4CL), exemplifying multilayered metabolic regulation (Bergelson et al., 2001; Asselin et al., 2015). Likewise, *Pantoea stewartii* infection attenuates photosynthetic networks while concomitantly inducing phenylpropanoid biosynthesis, suggesting a strategic diversion of carbon flux from growth to defense metabolite production. Proteomic mapping further demonstrates that the *Pyrenophora tritici-repentis* effector ToxA destabilizes the wheat photosystem II supercomplex, elevating ROS and accelerating necrotic symptom development (Manning et al., 2009). These multi-omics perspectives underscore the hierarchical coordination among transcriptional control, metabolic flux, and immune signaling, emphasizing the importance of direct metabolite profiling to resolve the functional phenotype of infection.

Advances in targeted and untargeted metabolomics enable high-resolution characterization of the metabolic architecture underlying plant–pathogen interactions (Hong et al., 2016; Ribbenstedt et al., 2018). Solvent polarity and instrument selection determine metabolome coverage: —aqueous extracts enrich for amino acids, sugars, and organic acids, whereas methanolic extractions captures specialized secondary metabolites (Cocuron et al., 2014, Cocuron et al., 2019). The combined application of GC-MS for volatile profiling and LC-MS/MS for semi-polar metabolite quantification provides detailed insight into defense-related metabolic reprogramming (Liu and Locasale, 2017). Metabolomics further clarifies how microbial effectors and toxins remodel host biochemistry to promote colonization. For instance, *Pseudomonas syringae*, deploys phytotoxin coronatine, hijacks the jasmonate receptor COI1, and disrupt SA–JA crosstalk, whereas *Ustilago maydis* suppresses SA biosynthesis to weaken maize defenses (Nomura et al., 2005; Geng et al., 2012; Toruño et al., 2016; Yang et al., 2017a). Comparative profiling shows that necrotrophs such as *Rhizoctonia solani* induce phenylacetic-acid-mediated necrosis and alter glutamate flux, whereas biotrophs such as *Candidatus Liberibacter asiaticus* reprogram phenylpropanoid and lignin metabolism in citrus (Milling et al., 2011; Li et al., 2019; Castro-Moretti et al., 2020). In nematode parasitism, *Heterodera schachtii* rewires amino-acid and hormone pathways—elevating ethylene, jasmonate, and proline—to form syncytial feeding sites (Labudda et al., 2016). Semi-biotrophs, including *P. syringae* and *Colletotrichum higginsianum*, exhibit dual-phase metabolic plasticity by secreting specialized effectors and terpenoid complexes that subvert host signaling and cellular integrity (McClerklin et al., 2018; Xin et al., 2018). Collectively, these integrative omics studies highlight the biochemical sophistication of pathogen strategies and metabolic flexibility underpinning plant immune resilience.

The advent of multi-omics integration has redefined systems-level analysis of plant–pathogen interactions, enabling identification of molecular signatures predictive of disease onset and resistance. Advanced computational frameworks—such as mixOmics, MOFA, and CWGCNA—integrate transcriptomic, proteomic, and metabolomic dataset, to uncover hierarchal relationship among gene expression, metabolic fluxes, and phenotypic outcomes (Rohart et al., 2017; Argelaguet et al., 2018; Liu, 2024). Within DIABLO mixOmics along with Bayesian latent factor models (e.g., MOFA2, iClusterPlus) applies sparse partial least squares and probabilistic inference to extract shared variance structures and infection perturbed regulatory nodes with precision. Network-oriented platforms such as Cytoscape, ReactomeGSA, and Pathway Commons integrate multi-omics data into dynamic molecular interaction networks revealing interdependencies among genes, proteins, and metabolites (Smoot et al., 2011; Griss et al., 2020). However, these integrative analyses remain computationally intensive because of high dimensionality, data heterogeneity, and complex normalization requirements (Schumann et al., 2025). High-performance and cloud-based infrastructures, — including Galaxy, Terra, or CyVerse—are essential for large-scale multi-omics workflows and deep-learning based feature extraction (Afgan et al., 2018; Li et al., 2020). Ultimately, advanced computation, experimental rigor, and interdisciplinary collaboration will transform metabolome-informed diagnostics and accelerate biomarker discovery in plant immunity (Balotf et al., 2025).

Pathogen perception triggers extensive transcriptional and metabolic reprogramming, unified by TF networks including WRKY, NAC, bZIP, and MYB that integrate MAPK, CDPK, and phytohormone signaling to recalibrate defense metabolism (Tsuda and Somssich, 2015; Zhang et al., 2016; Yuan et al., 2018). Multi-omics studies show that transcript levels often diverge from proteomic and metabolomic profiles, underscoring multi-layered post-transcriptional regulation of immunity (Feussner and Polle, 2015). Proteome-wide analyses identify modular defense architectures linking redox enzymes, cell wall modifiers, and chitosan-responsive proteins to pathogen resistance (Narula et al., 2020; Singh et al., 2020). Integrated transcriptome–metabolome analyses in barley and maize further reveal to co-regulated subnetworks that couple primary (amino acid, lipid, carbohydrate) and secondary (phenylpropanoid, flavonoid, phytohormone) metabolism with quantitative disease resistance (Yang et al., 2017b). Despite these advances, the plant immune metabolic network remains incompletely resolved and represents a key frontier for systems-level elucidation of defense-associated metabolic fluxes (Delplace et al., 2022).

## Structural and functional role of major metabolites bio-design in plants

The plant metabolome, comprising an estimated 200,000 to one million compounds is among the most chemically diverse in nature. Within this network, flavonoids illustrate how evolutionary innovation and enzymatic plasticity generate structural diversity and functional versatility. In *Arabidopsis. thaliana*, flavonols such as kaempferol and quercetin glycosides predominate, and accumulate under UV stress, while their biosynthesis is developmentally regulated through auxin transport modulation (Tohge and Fernie, 2016). In the Brassicaceae lineage-specific expansion of Phenylacyltransferase, further demonstrates how gene duplication drives metabolic diversification (Tohge et al., 2018). Similar catalytic innovations also underlie the biosynthesis of glucosinolates, terpenoids, saponins, and cyanogenic glucosides. Large enzyme super families, including cytochrome P450s and UDP-glycosyltransferases, enable combinatorial modification via glycosylation, methylation, and acylation, generating metabolite diversity that refines defense, signaling, and environmental adaptation (Bak et al., 2011; Thimmappa et al., 2014). Integration of transcriptomics, metabolomics, and genomics has accelerated pathway elucidation, by revealing regulatory networks in which subtle changes in enzyme specificity or substrate availability markedly metabolic flux and phenotype (Tohge and Fernie, 2010). Beyond structural diversification, metabolites display pronounced spatial and temporal patterning that shapes plants design and ecological resilience. Secondary metabolites including glucosinolates, phenylpropanoids, and flavonoids accumulate in organ-specific patterns, reflecting distinct physiological roles. In *A. thaliana*, glucosinolates concentrate in seeds and reproductive tissues, whereas indolic derivatives predominate in roots, establishing organ-level defense gradients (Reichelt et al., 2002). Phenylpropanoid intermediates such as cinnamic acid derivatives and coumarins are secreted in root exudates, to modulate rhizosphere signaling, whereas benzenoids such as salicylic acid couple redox balance with immune regulation. Flavonoid localization within the cytosolic, vacuole, and plastids is orchestrated by membrane-associated enzyme complexes, and glycosylation and acylation regulate intracellular transport, storage, and anthocyanic vacuolar inclusion formation (Zhao, 2015). Terpenoids, derived from isoprenoid precursors, exemplify adaptive metabolic deployment:—emitted volatiles such as β-ionone and 1,8-cineole mediate herbivore deterrence and inter organismic signaling (Dudareva et al., 2006). Collectively, these findings show that structural modifications, spatiotemporal compartmentalization, and evolutionary diversification of metabolites form a biochemical framework that enables plants to integrate development, defense, and environmental interactions (Wang et al., 2019). Plant metabolic versatility is also depending on extensive post-biosynthetic modifications that adjust metabolite stability, reactivity, and bioactivity in changing environments. Modifications such as glycosylation, methylation, acylation, and hydroxylation, — catalysed by transferase and cytochrome P450 super families, alter solubility, polarity, and volatility, thereby reshaping molecular signaling and metabolic homeostasis (Vaistij et al., 2009). In anthocyanins, sequential glycosylation and acylation regulate chromophore stability and vacuolar sequestration, with aromatic acylation (e.g., coumaroyl, and sinapoyl groups) enhancing pigment stability and aliphatic acylation (malonylation) increasing solubility and enzymatic resistance (Suzuki et al., 2002; Nakayama et al., 2003). Deglycosylation by β-glucosidases such as BGLU15 regulates flavonol turnover during stress recovery (Roepke and Bozzo, 2015). Beyond pigmentation, metabolite modification supports plant detoxification and self-tolerance: UDP-glucosyltransferases and cytochrome P450s conjugate xenobiotics to glycosyl or malonyl groups, facilitating vacuolar or apoplastic export (Gachon et al., 2005). In tomato, the steroidal glycoalkaloid α-tomatine illustrates toxicity control— as removal of lycotetraose residues markedly reduces membrane-disrupting activity (Ökmen et al., 2013). Likewise, glycosylated saponins and camalexin derivatives function as membrane-active phytoalexins whose modified forms balance antifungal potency with host cyto-protection (Kliebenstein, 2004). These dynamic chemical modifications act as precise regulators of plant fitness, by integrating defense chemistry with developmental and environmental plasticity. Metabolite modification also regulates hormone homeostasis, creating reversible reservoirs and modulating spatiotemporal signaling. Glycosylation of classical phytohormones—cytokinins, auxin, abscisic acid (ABA), and SA —modulates their bioavailability and stress responsiveness (Xu et al., 2012). In *Arabidopsis*, ZOG1-mediated cytokinin O-glucosylation reduces active zeatin pools, dampening morphogenetic signaling (Martin et al., 2001). Auxin conjugation via UGT84B1 or IAGLU produces IAA-glucose esters that buffer auxin during growth transitions (Korasick et al., 2013; Sauer et al., 2013). SA conjugation forms vacuolar SA 2-O-β-D-glucoside, hydrolysed during immune activation, while volatile methyl salicylate enhances systemic acquired resistance (Dean et al., 2005). ABA glucosylation, catalysed by ABA-GE synthases and hydrolysed by AtBG1 β-glucosidase, enables rapid hormone reactivation under dehydration stress (Lee et al., 2006). Collectively, these conjugation–deconjugation cycles act as molecular rheostats, linking metabolic flux to hormone perception and coordinating plant growth, defense, and environmental adaptation.

## Casting the engineering of metabolites and VOCs in plant resilience

Recent advances in modulating primary, and secondary metabolites, as well as volatile organic compound (VOCs) and metabolic pathways have transformed our understanding of plant metabolism and its potential for engineering crops with enhanced environmental resilience. This section explores the practical applications of specialize metabolites in strengthening plant stress tolerance.

## Plant primary and secondary metabolites as core determinants of plant immune function

**Figure 1:**
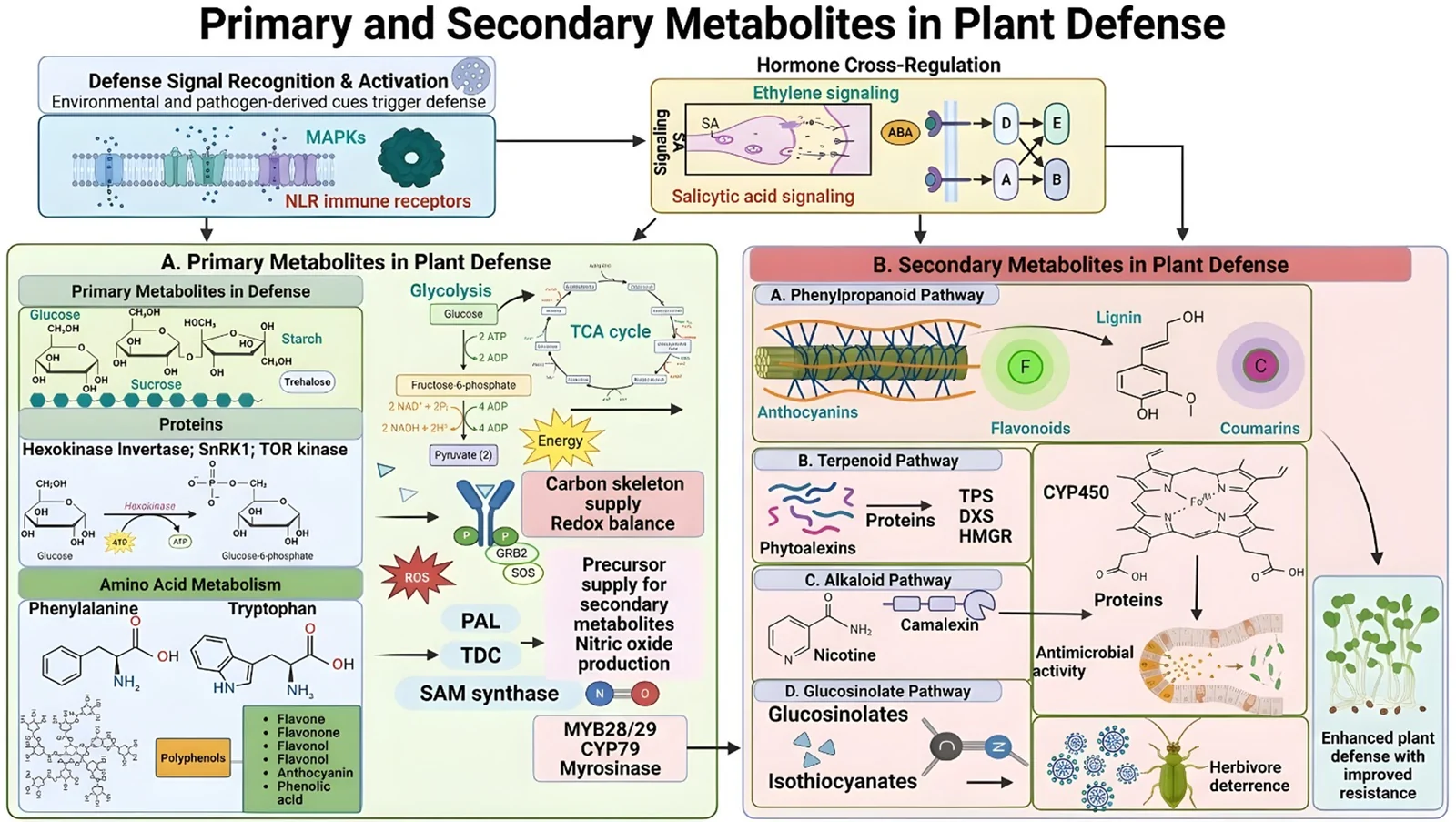
Integrated metabolic framework linking **(A)** primary and **(B)** specialized metabolism during plant defense responses. Pathogen recognition activates immune signaling networks, including mitogen-activated protein kinase (MAPK) cascades and nucleotide-binding leucine-rich repeat (NLR)-mediated immunity, which coordinate phytohormone signaling to reprogram primary metabolism and drive the biosynthesis of defense-associated specialised metabolites. Primary metabolic pathways, including carbohydrate metabolism, glycolysis, the tricarboxylic acid (TCA) cycle, and amino acid metabolism, provide the energy, reducing power, and metabolic precursors required for effective plant defense responses. CYP450, cytochrome P450 monooxygenase; ET, ethylene; MAPK, mitogen-activated protein kinase; NLR, nucleotide-binding leucine-rich repeat receptor; PAL, phenylalanine ammonia-lyase; SA, salicylic acid; TCA, tricarboxylic acid cycle; TPS, terpene synthase.

Primary metabolism is actively regulated determinant of plant immune competence, undergoing rapid and coordinated reprogramming after pathogen perception to meet the energetic, redox, and biosynthetic demands of defense activation (Bolton, 2009). MAMP- or effector-triggered immune induction elevates respiratory activity and drives large-scale transcriptional activation of central metabolic genes and pathways including octadecanoid-derived OPDA, reflecting increased requirements for ATP, reducing equivalents, and carbon skeletons necessary for defense protein synthesis, ROS production, and cell wall reinforcement (Heil and Bostock, 2002). Photosynthesis is locally downregulated at infection sites, not due to metabolic failure but as a strategic shift that reallocates assimilated carbon defense sinks while protecting the photosynthetic apparatus from oxidative damage generated during immunity (Berger et al., 2004; Bonfig et al., 2006). This source-to-sink transition is reinforced by cell-wall invertases and hexose transporters, which promote local sucrose cleavage and hexose accumulation to fuel defense metabolism while limiting apoplastic nutrient to pathogens (Bolton, 2009). Enhanced glycolytic flux and oxidative pentose phosphate pathway (PPP) activity further sustain immune responses by providing ATP and NADPH, the latter essential for NADPH oxidase driven oxidative bursts and SA–dependent defense signaling (Pugin et al., 1997; Frederickson Matika and Loake, 2014). Nitrogen metabolism is similarly restructured: amino acids serve as nitrogen carriers and substrates for energy pathways, while reactive nitrogen species such as nitric oxide link metabolic status to hypersensitive cell death and redox signaling (Besson-Bard et al., 2008). Under the oxidative and hypoxic conditions generated during immunity, alternative respiratory routes, including the γ-aminobutyric acid shunt, β-oxidation, glyoxylate cycle, and fermentative bypasses, are transiently recruited to maintain metabolic continuity and redox homeostasis when TCA cycle enzymes become constrained, as exemplified by Lr-34-mediated resistance (Bolton et al., 2008; Bolton, 2009). During immune activation, apoplastic sucrose cleavage and hexose uptake are closely coupled with enhanced glycolytic and oxidative pentose phosphate fluxes, enabling simultaneous ATP production and NADPH supply to fuel oxidative bursts, redox signaling, and downstream defense biosynthesis. Carbon flow through mitochondrial respiration integrates reducing equivalents from glycolysis, β-oxidation, and amino acid catabolism, thereby sustaining high energetic output and redox balance under defense-associated oxidative stress (Frederickson Matika and Loake, 2014). To buffer oxygen fluctuations and metabolic overload at infection sites, auxiliary routes such as the γ-aminobutyric acid shunt and pyruvate/2-oxoglutarate dehydrogenase bypass are transiently engaged, preserving carbon continuity and NADH/NAD^+^ homeostasis when the TCA cycle is constrained (Flaherty and Shelp, 2026). These interconnected metabolic pathways directly support callose deposition, ROS production, phenylpropanoid and shikimate pathway flux, phytoalexin synthesis, and pathogenesis-related protein accumulation, establishing primary metabolism as a prerequisite for effective immunity rather than a secondary consequence, as shown in **Figure 1a**. The major classes of defense-associated metabolites, their biosynthetic origins, immune functions, and metabolic bioengineering opportunities are summarized in **Supplementary Table 1**.

Moreover, primary metabolism–derived defense proteins form a conserved immune layer across viral, bacterial, and fungal patho-systems through direct antimicrobial activity and signal-responsive amplification (Zaynab et al., 2019). During viral infection, plants induce DRPs whose accumulation is tightly linked to SA signaling, hydrogen peroxide generation, polysaccharides (chitosan, laminarin and alginate) and virus-derived nucleic acids, enabling broad-spectrum antiviral activity. These primary metabolite–derived proteins function as early immune effectors, restricting viral replication and movement while reinforcing systemic resistance (Wang et al., 2012; Iglesias et al., 2015). Against bacterial pathogens, pathogenesis-related proteins including defensins, ribonuclease-like proteins, lipid-transfer proteins, and thionins exhibit direct antibacterial activity by targeting microbial membranes, nucleic acids, or nutrient transport, while amino acid metabolic reprogramming further supports immune reinforcement (Park et al., 2004). In fungal interactions, inducible antifungal proteins (β-1,3-d-glucan callose, polygalacturonase-inhibiting proteins (PGIPs), and ε-Viniferin) and hydrogen peroxide–generating enzymes disrupt pathogen cellular integrity and amplify host defense signaling (Terras et al., 1995; Ferreira et al., 2007). The inducible accumulation of defensins and oxidative proteins at infection sites shows primary metabolic outputs function both as energy-dependent defense investments and bioactive immune effectors that directly limit pathogen establishment across kingdoms.

As represented in **Figure 1b**, secondary metabolites form a chemically diverse, mechanistically potent layer that restricts pathogen through toxicity, structural reinforcement, and immune signal amplification (Bennett and Wallsgrove, 1994; Zaynab et al., 2018). Phenolics and lignin derivatives strengthen cell walls by limiting pathogen penetration while creating antimicrobial environments that disrupt microbial enzymes and membranes. Terpenes and alkaloids inhibit pathogen respiration, membrane integrity, and cellular signaling, providing broad-spectrum of defense against viruses (e.g., PVY, CMV and TMV via Bruceine-D), bacterial (via flavonoids), fungi (via α-tomatine), and herbivore, (via β-glucosidase and benzoxazinoids (BXs)-derived phytoanticipins unlocks biocidal aglycones) (Zaynab et al., 2018). Sulfur-containing metabolites, glucosinolates, and phytoalexins further enhance resistance by releasing reactive breakdown products that impair pathogen metabolism and colonization (Singh et al., 2023). At the molecular level, many secondary metabolites target a limited set of conserved pathogen processes, including enzyme catalysis, nucleic acid integrity, and reproductive machinery, thereby amplifying their effectiveness despite chemical diversity (Wink, 2003). Phenolic and polyphenolics, rich in reactive hydroxyl groups, destabilize pathogen proteins via hydrogen bonding and ionic interactions with sulfhydryl, amino, and hydroxyl moieties, inducing misfolding or conformational rigidity that disrupts protein–protein and protein–nucleic acid interactions. These cooperative noncovalent bindings impair enzymatic turnover and signaling fidelity, making secondary metabolites potent but selectively defense agents with low host toxicity.

**Figure 2:**
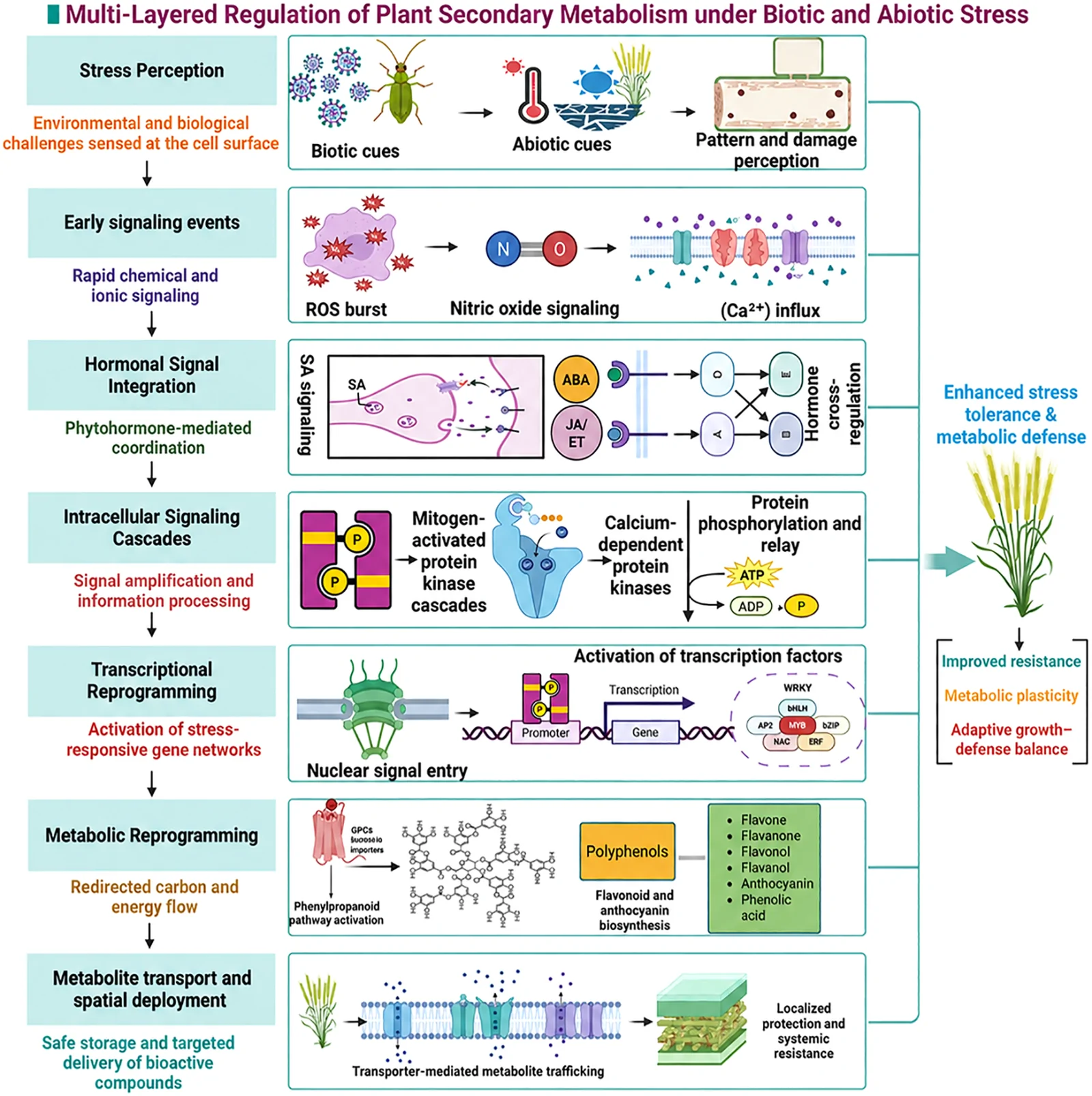
Multi-layered regulation of plant secondary metabolism under biotic and abiotic stress. Environmental and biological stress signals are perceived through pattern-recognition and damage-sensing systems, triggering early signaling events including reactive oxygen species (ROS) bursts, nitric oxide (NO) production, calcium influx, and phytohormone-mediated signaling. These responses activate intracellular kinase cascades, including mitogen-activated protein kinase (MAPK) pathways and calcium-dependent protein kinases (CDPKs), which relay stress information to the nucleus. Subsequent activation of stress-responsive transcription factors reprograms gene expression and redirects metabolic flux toward the biosynthesis of defense-associated metabolites, including phenylpropanoids, flavonoids, anthocyanins, and other antimicrobial compounds. Transporter-mediated trafficking and spatial sequestration facilitate the targeted delivery of metabolites to sites of infection and contribute to systemic resistance. Collectively, these regulatory layers integrate environmental perception, signaling, metabolism, and defense to enhance plant resilience, metabolic plasticity, and growth–defense balance. ABA, abscisic acid; CDPK, calcium-dependent protein kinase; ET, ethylene; JA, jasmonic acid; MAPK, mitogen-activated protein kinase; NO, nitric oxide; ROS, reactive oxygen species; SA, salicylic acid.

Modified secondary metabolites form an advanced defensive layer in which chemical diversification enhances both functional specificity and ecological effectiveness against pathogens and herbivores. Through enzyme promiscuity, gene duplication, and successive biochemical modifications, such as oxidation, glycosylation, methylation, acylation, and hydroxylation, plants convert primary precursors into structurally refined metabolites with optimized bioactivity, stability, and spatial deployment (Huang and Dudareva, 2023). These modifications adjust toxicity, volatility, solubility, and target selectivity, enabling metabolites to function as antimicrobials, feeding deterrents, signaling molecules, or priming agents across above- and below-ground tissues. Structural modification often decouples defense potency from constitutive toxicity, permitting inducible accumulation, controlled release, or compartmentalized storage that minimizes fitness costs while preserving rapid responsiveness. Enzyme promiscuity, gene duplication, and metabolite modification reactions expand chemical diversity from conserved primary metabolic precursors into lineage-specific defense compounds. The evolution and deployment of these specialized metabolites reflect a dynamic defense strategy in which metabolic plasticity, rather than compound abundance, determines the precision, durability, and adaptability of plant immune responses under biotic stress (Weng et al., 2021). Secondary metabolites diversify through a layered evolutionary process in which a limited set of primary metabolic precursors amino acids, nucleotides, isoprenes, and fatty acids—undergo iterative biochemical and genetic innovations to generate vast chemical repertoires (Huang and Dudareva, 2023). Enzyme promiscuity allows ancestral metabolic enzymes to accept structurally related substrates, generating new reaction outcomes without immediate genetic change, thereby seeding metabolic novelty under selective pressure. Subsequent metabolite modifications, including region-specific oxidation, glycosylation, methylation, and acylation, expand chemical space by fine-tuning molecular stability, reactivity, and cellular localization, allowing plants to balance bioactivity with self-tolerance (Birchfield and McIntosh, 2020). Gene duplication stabilizes advantageous chemistries by freeing redundant enzyme copies for sub- or neo-functionalization, creating lineage-specific metabolic branches optimized for distinct ecological challenges. These mechanisms drive a unidirectional expansion from conserved primary metabolism to secondary defense chemistries, demonstrating that metabolic diversity in plant immunity is an evolutionarily structured outcome of biochemical flexibility and genetic innovation. The phenylpropanoid pathway, exemplifies this complexity, forming a tightly regulated metabolic networks central to plant immunity. Initiated by phenylalanine ammonia lyase (PAL) and refined through cinnamate 4-hydroxylase (C4H) and 4-coumarate-CoA ligase (4CL), this pathway generates activated intermediates that feed flavonoid biosynthesis via chalcone synthase (CHS) and chalcone isomerase (CHI), forming a central branching node for metabolic diversification (Birchfield and McIntosh, 2020). Downstream enzymes including flavone synthase (FNS), flavanone 3-hydroxylase (F3H), flavonol synthase (FLS), dihydroflavonol 4-reductase (DFR), and anthocyanidin synthase (ANS) generate structurally and functionally distinct flavonoid scaffolds such as flavones (e.g., apigenin), flavonols (e.g., kaempferol), anthocyanidins (e.g., pelargonidin), and proanthocyanidins (e.g., leucopelargonidin) contributing to cell wall reinforcement, NO, ROS scavenging, UV protection, and antimicrobial defense pathways (CDPK and MAPK). Its modularity, cofactor dependence (e.g., NADPH, Fe²^+^, 2-oxoglutarate), and tissue-specific regulation make the pathway an ideal target for defense-oriented engineering that enhances immunity without disrupting core metabolism, as depicted in **Figure 2**.

Beyond angiosperms, bryophytes employ a conserved strategy linking immune-associated terpenoids and very-long-chain fatty acids (vlcFAs) to central metabolic fluxes derived from glycolytic, the TCA cycle, and Calvin–Benson–Bassham (CBB) cycle intermediates, with plastidial and cytosolic acetyl-CoA pools fueling both methylerythritol phosphate (MEP)- and mevalonate (MVA)-derived [isopentenyl pyrophosphate (IPP)/dimethylallyl pyrophosphate (DMAPP)] terpenoid biosynthesis (Lindström Battle and Sweetlove, 2025). In woody perennials, resinous monoterpenes and diterpenes function as chemical deterrents and physical barriers, while trichome-stored O-acyl sugars impair insect respiration and mobility. The cuticle also integrates hydrocarbons and phenolics providing persistent antimicrobial protection. Direct chemical defense can be constitutive or inducible, with phytoalexins such as camalexin and sakuranetin synthesized *de novo* under WRKY, MYB, and ERF transcriptional control. Chromatin dynamics, including H3K4me3 enrichment, chromatin re-modeling, and non-coding RNA regulation, are increasingly recognized as key determinants of the spatial and temporal precision of specialized metabolite deployment during immune responses (Bai et al., 2024). The evolution of plant secondary metabolites reflects progressive optimization through lineage-specific enzymatic innovation, enabling plants to balance developmental constraints with ecological defense demands (Xu and Gaquerel, 2025). Ancestral metabolites often exhibit intrinsic cytotoxicity, selecting for modifications that attenuate self-toxicity while preserving or enhancing defensive efficacy (Erb and Kliebenstein, 2020). Recurrent recruitment of cytochrome P450 monooxygenases and UDP-glycosyltransferases diversifies core scaffolds through region-specific oxidation and glycosylation, generating modular metabolite systems that function as direct toxins, inducible defense, or volatiles mediating tritrophic interactions (Wisecaver et al., 2017).

## Targeted and untargeted pathway engineering as complementary routes to resilience

Targeted and untargeted pathway engineering represent complementary strategies that, together, shift plant defense from single-metabolite amplification to resilient metabolic reprogramming. Targeted approaches excel when pathway topology, control points, and regulatory levers are well defined allowing intentional flux steering via enzyme tuning, transcriptional control, compartmental routing, or hormone-coupled induction. Untargeted approaches provide the breadth to reveal hidden metabolic sinks, compensatory routes, and cost-driving perturbations that are difficult to predict. In practice, resilience emerges when precision edits are guided by discovery-grade metabolome profiling, ensuring defense chemistry is elevated, properly timed, spatially deployed, and metabolically sustainable under repeated or mixed biotic challenges (Yan et al., 2022; Han et al., 2023). Targeted engineering thus functions as a mechanistic control layer that fortifies well-characterized defensive branches. For example, phytoalexin engineering has shifted from constitutive overexpression to inducible architectures that match infection kinetics and local patterns. Defense metabolites such as camalexin, sakuranetin, and stilbenes are most effective when rapidly mobilized at invasion sites, coordinated with upstream signaling and transcriptional relays (e.g., WRKY- and ERF-centered programs), and buffered against self-toxicity through conjugation, sequestration, or transport (Jeandet et al., 2014). Epigenetic regulation and chromatin-mediated priming further enhance this rapid-on/rapid-off logic responsiveness as demonstrated by MEJA (Methyl jasmonate) mediated activation of *OsBBPI* and *OsPOX* across developmental stages (Laura et al., 2018).

Another canonical example of targeted intervention lies in engineering the shikimate axis, which operates as a high-leverage metabolic backbone providing aromatic and hexanoic precursors for phenylpropanoids, flavonoids, lignin-associated barriers, and SA-linked immunity (Aranega-Bou et al., 2014). The principal challenge is not simply increasing shikimate flux, but preserving network stability under feedback inhibition and precursor competition. Studies targeting rate-limiting steps such as 3-deoxy-d-arabino-heptulosonate-7-phosphate synthase (DAHPS) and downstream branch points show that coordinating precursor expansion with downstream demand, prevents carbon accumulation in inactive intermediates and avoids excessive drain on primary metabolism (Tzin and Galili, 2010). Plants achieve this through metabolic channeling, dynamic enzyme assemblies, and transporter-mediated partitioning directing shikimate-derived intermediates toward defense while minimizing autotoxicity, an architectural principle that engineering strategies should emulate (Tohge et al., 2013; Zhang and Fernie, 2021). Hormone-centered engineering provides an integrative leverage point that naturally bridges targeted and untargeted strategies. JA and SA pathways act as both upstream signaling switches and metabolic allocation frameworks coordinating carbon, nitrogen, and reducing power allocation between growth and defense (Roychowdhury et al., 2024). Since SA and JA often act antagonistically, resilience rarely results from maximizing a single hormone. Instead, effective defense arises when promoter logic, transcriptional integration (e.g., NPR1, ORA59 and MYC2-associated modules), and inducible signal decoding restrict activation of defense metabolism to relevant biotic cues, thereby minimizing chronic growth penalties (Van der Does et al., 2013). Programmable promoters (synthetic promoter-D box-D box (SP-DD), synthetic promoter-F element-F element (SP-FF) and synthetic promoter-F element-F element-D box-D box (SP-FFDD)) responsive to combined stress signals, alongside genome editing to counter effector-mediated suppression of immune signaling, exemplify how targeted modifications improve durability by aligning with hormonal dynamics (Shokouhifar et al., 2019; Yasmeen et al., 2023).

Engineered overexpression of biosynthetic genes for compounds such as camalexin in *A. thaliana* or glyceollin in Glycine max enhances localized chemical defense (Monsalvo et al., 2024). While, reprogrammed lignin and callose biosynthesis strengthen cell walls to restrict pathogen ingress (Weng and Chapple, 2010; Monsalvo et al., 2024). Manipulating hormonal signaling particularly SA, JA, and ethylene remains central to defense amplification. Genetically enhancing SA biosynthesis or sensitivity confers broad-spectrum resistance to biotrophs, whereas modulating JA and ethylene pathways strengthens defense against necrotrophs and herbivorous insects (Glazebrook, 2005; Bodenhausen and Reymond, 2007; Pangesti et al., 2016). For example, engineering AtICS1 (Isochorismate Synthase 1), enhances SA-mediated systemic acquired resistance in *Arabidopsis* and tobacco (Zeier, 2021). TF (NHP, EDS1 and SARD) and SA act as key regulatory nodes for orchestrating defense gene networks (Hartmann and Zeier, 2019). While, WRKY33/70 integrates SA- and JA-dependent signals to coordinate defense gene expression upon pathogen recognition (Li et al., 2004; Zheng et al., 2006). Overexpression of WRKY33 enhances camalexin biosynthesis via PAD 3-1 and confers resistance to necrotrophic fungi, while AtMYB12 promotes flavonoid biosynthesis in *Nicotiana tabacum*, improving antimicrobial metabolite accumulation and pathogen resistance (Ferreira et al., 2007). These regulatory circuits are increasingly programmable via synthetic biology tools, including synthetic TF and dCas9-based activators that induce multi-gene defense modules without imposing fitness costs (Feng et al., 2013). Targeted editing of phenylpropanoid, alkaloid, flavonoid, and benzoxazinoid pathways has yielded robust resistance across crops (Zhou et al., 2022b). Upregulating phenylpropanoid genes enhances lignin, coumarin, and stilbene accumulation, strengthening antimicrobial activity and vascular integrity under pathogen pressure (Ninkuu et al., 2025). In cereals, benzoxazinoid engineering improves resistance to *Fusarium graminearum*. CRISPR/Cas strategies, including NPR1 repressor modification or deletion of glycosyltransferases boost pathogenesis-related gene expression and bioactive aglycones, reinforcing basal immunity and antifungal potency (Chong et al., 2002; Zhao et al., 2025). Despite careful targeting, engineered pathways often underperform due to intermediate diversion, inactive storage, or spatial misrouting away from infection sites. Untargeted metabolomics, therefore, serve as critical discovery layer, revealing flux leakage, unintended derivatization, and compensatory rerouting that pathway blueprint alone cannot predict. For example, endogenous glycosyltransferases can fragment flux into competing products, such as pinoresinol and isopentenyl diphosphate (IPP) from mevalonate 5-phosphate (MVAP), feruloyl-CoA from caffeic acid through caffeoyl-CoA or ferulic acid in the phenylpropanoid pathway or ajmaline from acetylnorajmaline through norajmaline or acetylajmaline in the monoterpenoid indole alkaloids); highlighting dominant diversion chemistries first exposed by metabolomic profiling of targeted/untargeted pathways (Jones and Vogt, 2001; Shih and Morgan, 2020). Increasingly, untargeted analysis are combined with quasi-targeted acquisition modes (SIM/MRM) to maintain quantitative robustness while capturing broad metabolite space, critical when defense phenotypes arise from coordinated shifts across multiple compounds rather than a single marker (Rampler et al., 2021; Panzenboeck et al., 2024).

Untargeted discovery becomes more actionable when integrated with systems biology, as resilience emerges at the network-level. Genome-scale metabolic reconstructions and constraint-based simulations translate broad metabolomic shifts into testable hypotheses on bottlenecks, alternative routes, and resource trade-offs, particularly in compartmentalized plant systems. Early Arabidopsis models link genotype-to-capacity, while subsequent expansions to photosynthetic and crop systems, revealed control points hidden by network complexity (de Oliveira Dal’Molin et al., 2010). Recent approaches highlight regeneration-coupled immunity: inducible ZmWUS2–ZmBBM expression in *Sorghum bicolor* restores defense-competent cells synchronizing local metabolite production with systemic immune priming and conferring resistance to *Colletotrichum sublineola/gloeosporioides* (Haque et al., 2018; Yan et al., 2023; Xavier et al., 2026). Reactivation of patterning regulators such as ZmPLT1 and ZmCUC2 establishes embryonic transcriptional states that coordinate with WRKY-driven antimicrobial biosynthesis. In *Medicago truncatula* and *Oryza sativa*, PLT5 and STM1 similarly induce callus competence while activating PRRs and defense genes, revealing a conserved link between cellular plasticity and immune metabolism (Ikeuchi et al., 2019). Synthetic scaffolds, dynamic enzyme colocalization, and metabolic channeling further minimizes crosstalk and increase substrate transfer efficiency. Coupled with flux balance analysis and genome-scale modeling, these approaches identify rate-limiting steps and enable rational, spatially and temporally precise reconfiguration of defense metabolism. In plant-pathogen interactions with *Xanthomonas campestris* pv. campestris (Xcc) glycolysis and the Entner–Doudoroff pathway serve as a primary catabolic routes with PPP flux, contributing to resistance via both antimicrobial accumulation and strategic restriction of pathogen nutrient access (Schatschneider et al., 2014). Genome-scale reconstructions of *Ralstonia solanacearum* indicate that depriving invaders access to essential metabolites or exopolysaccharides can dirupt early infection programs more effectively (Peyraud et al., 2016). Combining targeted and untargeted strategies enables a disciplined design–build–test cycle: discovery-driven metabolomics defines actual metabolic states; modeling prioritizes interventions with maximal leverage; targeted engineering implements coordinated push–pull control, enzyme routing, transporter modulation (e.g., hyoscyamine, berberine via NtJAT1 and NtJAT2, and vitamin B6 via NtNUP1), and inducible regulation; and validation confirms enhanced defense without chronic stress (Xiao et al., 2025). In this framework, resilience emerges not from metabolite abundance alone but from precise spatiotemporal orchestration of metabolic networks. The representative metabolic bioengineering strategies, their experimental validation status, translational readiness, practical applications, and current limitations are outlined in **Supplementary Table 2**, providing an evidence-based overview of the maturity and future potential of programmable metabolic engineering approaches for strengthening plant immunity. These integrated strategies redefine plant metabolic defense as a programmable, systems-level trait, encouraging the development of climate-resilient crops with durable immunity and minimal ecological cost.

## Metabolic modeling and VOC in plant defense

Genome-scale metabolic models (GEMs) have evolved from static stoichiometric reconstructions into multi-layered system frameworks that integrate gene expression data, enzymatic capacity constraints, and macromolecular biosynthesis requirements to simulate intracellular metabolite fluxes with increasing physiological realism (Kim et al., 2017). Advanced hybrid models, such as resource balance analysis and expression-constrained flux models, enable more accurate prediction of metabolite uptake rates, secretion fluxes, and VOC emission dynamics under stress conditions (Blonde et al., 2025). These approaches quantitatively resolve metabolic trade-offs, including carbon and nitrogen allocation between macromolecule biosynthesis and defense-associated VOC production, which are critical during pathogen and herbivore challenge. Regulatory network-augmented GEMs further reveal phenotypic switching and transcriptional reprogramming that govern defense-associated metabolite routing. Integrating protein synthesis and degradation dynamics enhances prediction of VOC precursor availability, while macromolecule-constrained models resolve secretion–proliferation trade-offs essential for spatially coordinated VOC deployment (Lerman et al., 2012). At the organ scale, multi-organ metabolic models bridge localized biosynthetic hotspots with whole-plant systemic VOC emission, offering predictive resolution of defense priming, long-distance signaling, and growth-productivity trade-offs (Gomes de Oliveira Dal’Molin et al., 2015; Blonde et al., 2025). Recent modeling innovations incorporate substrate uptake dynamics and protein synthesis costs via metabolism and expression models and their thermodynamically constrained successors (ETFL), thereby improving flux prediction accuracy while enhancing computational efficiency (Salvy and Hatzimanikatis, 2020). Resource Allocation Models (RAMs) further unify enzyme cost and protein turnover dynamics, albeit they entail greater model construction and parametrization complexity (De Becker et al., 2022; Schroeder et al., 2024). Moreover, integrating macromolecule biosynthesis and secretion modules enables quantitative estimation of the energetic costs associated with effector deployment and cell wall degradation in phytopathogens, thereby capturing previously underrepresented mechanistic layers of plant–microbe interactions (Peyraud et al., 2019).

To extend the modeling framework beyond cellular resolution, organ-centered and multi-organ metabolic models have been developed to resolve spatial metabolic partitioning and coordinate systemic flux distribution across tissues (Poolman et al., 2009, Poolman et al., 2013). Organ-centered models simulate discrete metabolic activities within individual tissues by incorporating compartment-specific constraints and explicit transport interfaces (Dal’Molin et al., 2010). Multi-organ models extend this framework by linking tissue-level metabolic networks through shared metabolite pools, thereby enabling quantitative analysis of inter-organ flux distribution under dynamic conditions. For example, dynamic flux balance approaches (dFBA) applied to *Arabidopsis thaliana*, maize, tomato, and *Medicago truncatula* elucidates diel nitrogen allocation patterns, characterized by nocturnal root storage is followed by diurnal remobilization to the shoot (diCenzo et al., 2020). Recent advances in GEM enabled predictive mapping of plant–microbe metabolic interfaces across phyllosphere and rhizosphere compartments (Blonde et al., 2025). Integrating GEMs for diverse *A. thaliana*-associated PGPR strains enables inference of microbial cooperation–competition dynamics and community organization (Schäfer et al., 2023). Multi-organ metabolic models of soybean coordinated with *Bradyrhizobium diazoefficiens* GEMs enable quantitative simulation of nitrogen fixation efficiency and its agronomic trade-offs against fertilizer input (Holland et al., 2023). In parallel, embedded host GEMs with *Phytophthora infestans* models reveal nutrient diversion strategies employed by pathogenic oomycetes during infection, highlighting host-derived branched-chain amino acids (BCAAs) as critical for virulence gene expression (Rodenburg et al., 2019). Moreover, ML-constrained ensembles of *Pseudomonas syringae* GEMs, guided by planta transcriptomics, have been used to identify immune-imposed bottlenecks in pathogen colonization (Tubergen et al., 2023). These cross-domain metabolic integrations spotlight the potential of GEM frameworks to predict microbial behavior under plant-imposed selective pressures and equip dynamic flux allocation modeling across holobiont interfaces. Recent ML integration with systems biology is advancing the predictive accuracy of metabolic models, particularly through deep learning approaches that infer catalytic rate constants (Kcat) and unified kinetic parameters (UniKP) from enzyme–substrate features, thereby improving enzyme-constrained GEMs (Kroll et al., 2023; Yu et al., 2023a). ML has been instrumental in identifying novel immune regulators, pathogen effectors, and interaction targets in plant–pathogen systems (Mishra et al., 2019), while also enhancing constraint-based models through the refining model outputs and gap-filling of incomplete datasets (Zampieri et al., 2019).

VOCs are synthesized from central metabolic precursors—acetyl-CoA, pyruvate, phosphoenolpyruvate (PEP), and erythrose-4-phosphate (E4P)—which channel into four major primary biosynthetic pathways: the mevalonate (MVA), methylerythritol phosphate (MEP), shikimate/phenylalanine, and lipoxygenase (LOX) pathways (Chen and Liao, 2025). The MVA and MEP pathways converge at isopentenyl pyrophosphate (IPP) and dimethylallyl pyrophosphate (DMAPP), which are subsequently converted into mono-, sesqui-, and diterpenes via prenyl intermediates including geranyl (GPP), farnesyl (FPP), and geranylgeranyl pyrophosphate (GGPP). In parallel, the shikimate pathway, originating from PEP and E4P, directs flux toward phenylpropanoid and benzenoid biosynthesis through phenylalanine, while the LOX pathway oxidizes linoleic and linolenic acids generate green leaf volatiles (GLVs) and jasmonate derivatives (Maeda and Dudareva, 2012). These biosynthetic axes function in a coordinated, tightly regulated manner, enabling spatially and temporally precise production of structurally diverse VOCs.

Terpenoid VOC biosynthesis is partially partitioned between the cytosolic MVA and plastidial MEP pathways, both of which generate the universal C5 isoprenoid precursors IPP and DMAPP (Dudareva et al., 2013). The MEP pathway comprises five enzymatic steps, converting MEP into IPP and DMAPP utilizing pyruvate (Pyr) and glyceraldehyde-3-phosphate (GAP) sourced from glycolysis and the PPP. However, plastidial glycolytic flux is limited due to low phosphoglycerate mutase and enolase activity, making plastidial Pyr supply dependent on cytosolic import. Disrupting the Pyr transporter in *A. thaliana* reduces plastidial IPP biosynthesis, demonstrating cytosolic Pyr is a limiting substrate for isoprenoid flux (Bayer et al., 2011; Furumoto et al., 2011). Short-chain prenyltransferases convert IPP and DMAPP into GPP, FPP, and GGPP providing substrates for terpene synthase (TPS) and cyclase to produce monoterpenes, sesquiterpenes, diterpenes, and irregular terpenes such as geranyllinalool (Karunanithi and Zerbe, 2019). This hierarchical organization ensures precise metabolic control of carbon flux allocation toward specialized VOC biosynthesis. Functionally, VOCs serve as defense signals with multiple ecological roles. GLVs, including, (Z)-3-hexenal and (Z)-2-hexenol are rapidly released following wounding or herbivory, and their emission profiles can be modulated by insect-derived elicitors to attract predators and parasitoids. Terpenoid volatiles, such as β-caryophyllene mediate rhizosphere recruitment of entomopathogenic nematodes and attract parasitic wasps in aerial tissues. Notably, β-caryophyllene exhibits ecological plasticity, function as an antimicrobial compound in *Arabidopsis* and as a root-emitted signal in *Zea mays* (Frey et al., 1997).

**Figure 3:**
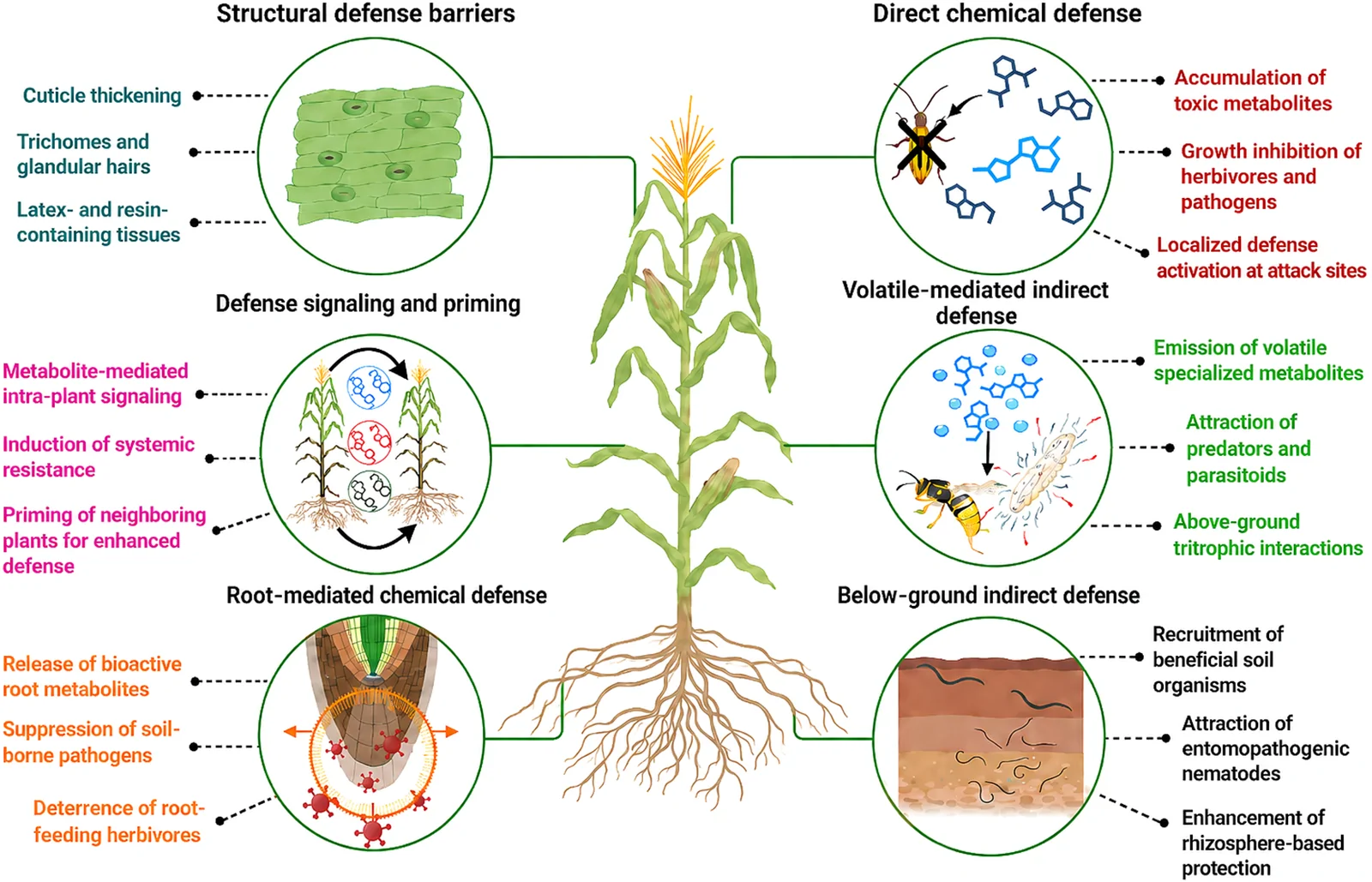
Roles of plant secondary metabolites in direct and indirect defense responses. Plant secondary metabolites contribute to defense through multiple complementary mechanisms operating above and below ground. Direct defense includes structural barriers, toxic metabolites, antimicrobial compounds, and anti-herbivore chemicals that inhibit pathogen establishment and pest feeding. Indirect defense is mediated through volatile organic compounds (VOCs) and root-derived metabolites that recruit beneficial organisms, attract natural enemies of herbivores, promote rhizosphere protection, and induce systemic resistance. Metabolite-mediated signaling further facilitates intra-plant communication, defense priming, and coordination of local and systemic immune responses.

The biochemical basis for VOC diversity arises from enzyme promiscuity, scaffold modification, and gene duplication. Broad-substrate TPS, methyltransferases, and glycosyltransferases catalyze versatile reactions, exemplified by two *Arabidopsis* TPS enzymes that produce the majority of floral sesquiterpenes. Methyltransferases from *Stephanotis floribunda* and *Nicotiana suaveolens* produce a range of methyl esters from cinnamic and benzoic acids (Huang et al., 2022). Scaffold modification via cytochrome P450s, BAHD acyltransferases, methyltransferases, and glycosyltransferases diversifies the chemical properties, subcellular localization, and stability of VOCs (Chu et al., 2011). At the regulatory level, targeted modulation of transcription factors and epigenetic switches enhances biosynthetic capacity. Master regulators, including WRKY33, MYB51, ERF1, and ORCA3 activate core biosynthetic genes involved in phytoalexins, camalexins and terpenoids production (Mora-Vásquez et al., 2022; Zhou et al., 2022a). Chromatin modifiers such as HDA6 and HDA9 regulate promoter accessibility via histone deacetylation, thereby dynamically modulating gene expression during pathogen stress (Tao et al., 2023). Engineering JA/SA-responsive promoters together with CRISPR-mediated editing of effector targets further strengthen inducibility and resistance specificity (Bauters et al., 2021; Molla, 2022). Long noncoding RNAs and JA-responsive chromatin looping establish amplification circuits, that increase the output of key defense components including lipoxygenase (LOX), catalase (CAT) antimicrobial peptides (AMP) (Huang et al., 2023). Metabolic bio-engineering of the shikimate, terpenoid, alkaloid, and phenylpropanoid pathways has increased volatile and non-volatile defense metabolites. Overexpression of PAL (phenylalanine ammonia lyase), TPSs (terpene synthase), and strictosidine synthase, combined with compartment-specific gene stacking and flux channeling, has driven overproduction of compounds such as linalool, thymol, (E)-β-caryophyllene, and methylated benzenoids (Beran et al., 2019; Koeduka, 2024). Co-expression with cytochrome P450s enzymes enables combinatorial biosynthesis of structurally novel antifungal metabolites, including 2-benzoxazolinone with activity against *Fusarium species* (Gao et al., 2022). Tissue-specific targeting of biosynthesis to glandular trichomes, laticifers, and secretory cavities coordinated through membrane transporters, glycosyltransferases, and tailored promoters enhances both accumulation and release of labile volatiles (Dixon and Dickinson, 2024). Also, VOCs function as airborne priming cues in direct and indirect defenses, enhancing a defense readiness in neighboring or distal tissues, as shown in **Figure 3**. This priming involves enhanced immune signaling capacity, and accelerated transcriptional responsiveness (Mauch-Mani et al., 2017). The shikimate pathway constitutes a central metabolic framework for the biosynthesis of the aromatic amino acids phenylalanine, tyrosine, and tryptophan, and their specialized derivatives including SA, flavonoids, and lignin precursors (Shende et al., 2024). Strategic overexpression of key shikimate pathway enzymes including DAHPS and chorismate mutase enhances chorismate availability, thereby elevating defense-associated secondary metabolites downstream of enolpyruvylshikimate 3-phosphate (EPSP) and conferring broad-spectrum resistance to fungal and bacterial pathogens (Tzin and Galili, 2010). Localized activation of the phenylpropanoid branch, facilitated by tissue-specific promoters, permits infection-site–specific metabolite allocation while minimizing autotoxicity. Recent studies reveal that plastidic and cytosolic transporters regulate precursors flux into phenylpropanoid biosynthesis, and feedback-insensitive phosphate transporters can be exploited to enrich shikimate-derived metabolic throughput (Dixon, 2001; Tzin and Galili, 2010).

Complementarily, JA and SA function as antagonistic yet convergent hormonal hubs that reprogram defense metabolism in a context-dependent manner. SA primarily trigger SAR and biotrophic pathogen defense, whereas JA modulates inducible responses to necrotrophs and insect herbivory (Zhang et al., 2025b). Metabolic rewiring under these hormones is coordinated by transcriptional integrators including NPR1 and MYC2, which target hormone-responsive cis-regulatory modules. Precision engineering strategies employing JA-SA dual-inducible promoters enable defense activation exclusively in response to relevant biotic stress, minimising metabolic costs during normal growth (Li et al., 2023). Additionally, CRISPR/Cas-mediated disruption of effector-targeted host genes strengthens hormonal robustness, while long noncoding RNAs and epigenetic regulators such as HDA6 and HDA9 reinforce transcriptional memory and amplify defense output through histone remodeling (Tao et al., 2023). Transcription factors such as PtMYB14 regulate complex networks that control terpene and flavonoid biosynthesis and contribute to vascular differentiation (Guan et al., 2026). However, trade-offs must be managed particularly the growth-inhibitory effects of sustained JA activation via gibberellin antagonism (Panda et al., 2022). Collectively, these strategies constitute a modular framework for engineering volatile defense traits with ecological precision and metabolic efficiency.

## Emerging tools for programmable metabolic bioengineering

**Figure 4:**
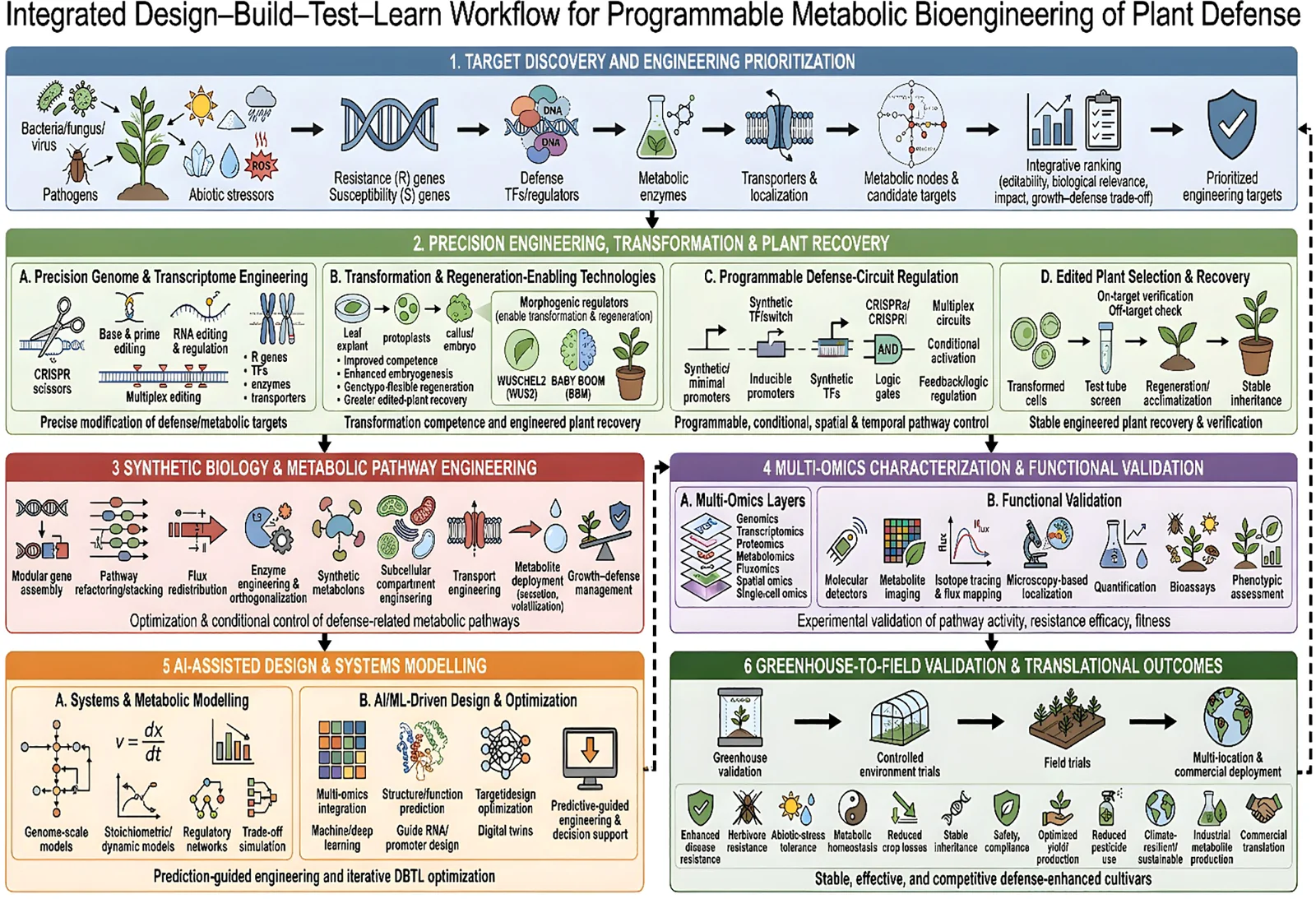
Integrated workflow of programmable metabolic bioengineering for plant defense. The schematic illustrates a sequential Design–Build–Test–Learn framework for developing defense-enhanced crops through programmable metabolic bioengineering. The workflow encompasses (i) target discovery, (ii) precision genome engineering, (iii) synthetic pathway design, (iv) multi-omics validation, (v) AI-assisted optimization, and (vi) greenhouse-to-field translation for the advancement of resilient crops. AI, artificial intelligence; BBM, BABY BOOM; CRISPR, Clustered Regularly Interspaced Short Palindromic Repeats; GEMs, genome-scale metabolic models; WUS, WUSCHEL2.

Plant metabolomics has entered in a new era as a transformative technology in plant science, enabling comprehensive reprogramming of biological systems through metabolite profiling, targeted metabolite analysis in physiological processes, and understanding previously uncharacterized biosynthetic pathways. Here, we discuss current approaches with potential applications, placing special emphasis on metabolic modeling regarding genome editing, multi omics-technologies and artificial intelligence (AI) driven metabolomics, illustrated in **Figure 4**.

## Contemporary tools used in metabolic bioengineering

The expansion of CRISPR/Cas systems has significantly enhanced the bet-hedging capacity of metabolic bioengineering by increasing the range of precise molecular tools available for targeted manipulation of plant genomes (Osakabe et al., 2018; Tan et al., 2023). Class 1 CRISPR systems, including the Cascade-Cas3 complex, employ multi-protein effector complexes that mitigate long-range DNA degradation. In contrast, Class 2 nucleases, which include Cas9, Cas12, and Cas13, function as single protein effectors and provide greater ease-of-use and versatility in designing constructs for metabolic bioengineering (Wada et al., 2022; Liu et al., 2025a). Consequently, Cas9 remain one of the primary enzymes utilized for plant genome editing: however, Cas9 variants, such as SaCas9 and ScCas9, expanded targeting capabilities by recognizing additional protospacer adjacent motif (PAM) sequences beyond the canonical NGG motif. Additionally, directed evolution has produced PAM relaxed nucleases, such as Cas9-NG, SpG and the nearly universally targetable SpRY, enabling modification at most genomic loci and thereby reducing the probability of a plant escaping modification due to compensatory mutations (Abudayyeh et al., 2016; Cox et al., 2017). Cas12 nucleases offer an advantage over Cas9-based systems, because they recognize AT-rich PAM sequences cleave DNA to generate staggered double strand breaks, allowing for homologous directed repair. Cas12 also processes its own crRNA, and enabling multiplex arrays for simultaneous editing of multiple loci, and decreasing the time required complex genetic modifications (Osakabe et al., 2020). Variants, such as LbCpf1-RR and LbCpf1-RVR, further expand the range of editable genomic loci. Ultra-compact Cas12f nucleases (e.g. OsCas12f1 and RhCas12f1) which are less than one-third the size of SpCas9, help in overcome delivery constraints in system where vectors capacity limits the efficient delivery of gene editing enzymes. Cas13 effector systems provide an additional independent mechanism for regulating RNA expression levels. Cas13 variants such as Cas13d, Cas13x and CasRx enable reversible transcript-level regulation without permeant genome modification, thereby facilitating transient regulatory feedback circuits that assist stable engineered traits under fluctuating environmental conditions (Mahas et al., 2019; Xu et al., 2025). To level-up the effectiveness of this approach, efficient delivery strategies can be combined with viral-based CRISPR-Cas9 delivery systems to enhance metabolic and genomic engineering outcomes (Ma et al., 2020). Geminivirus replicon-based systems can amplify editing components via rolling circle replication, enabling high levels of transient genome editing in crops traditionally considered recalcitrant, while minimizing cytotoxic effects associated with alternative editing platforms (Baltes et al., 2014). Overall, combining the diversity of nuclease types (from SpCas9 to Cas9, Cas12, and Cas13 variants), along with replicon-based delivery systems exemplifies bet-hedging strategy, where multiple nucleases, substrates, and delivery platforms ensure robust control of metabolic pathways even individual components fail. An early demonstration of the bet-hedging principle in multiplex genome editing (MGE) came from initial TALEN studies, showing the feasibility of targeting polyploid genomes and multi-gene families to edit mildew resistance genes in wheat or perform large scale glycoengineering in *N. benthamiana* (Li et al., 2013b). TALENs have also been employed to edit >100 copies of caffeic acid O-methyltransferase in sugarcane reducing lignin content and enhancing saccharification efficiency (Kannan et al., 2018). While TALENs require custom DNA-binding domains and their TAL-Eco’s efficiency decreased with increasing gene family size, CRISPR/Cas system offer greater scalability than TALENs enabling multiplex editing with a single nuclease guided by multiple sgRNAs. Golden Gate Assembly, ribozyme-processed polycistronic array and tRNA processing systems, refines the expression of dozens of sgRNAs from a single construct, allowing simultaneous editing of multiple, unrelated genomic loci with unprecedented ease (Liu et al., 2025a). This capability has also fundamentally improved the role of CRISPR in metabolic engineering by providing researchers with a stratagem to overcome genetic redundancy of polyploid crops or complex multigene biosynthetic networks.

The precision frontier has been advanced via base editing and prime editing. Cytosine base editors (CBEs) and adenine base editors (ABEs) catalyze predictable transition mutations (C•G → T•A, A•T → G•C), while engineered TadA8e deaminases generate highly specific products with a minimal bystander edits (Richter et al., 2020). Dual editors such as A&C-BEmax allow cytosine and adenine editing; however, their viral delivery is often limited by spatial constraints. Compact dual systems, such as TadA-dual, mitigate these limitations, while preserving multiplex substitutions capabilities (Gaudelli et al., 2017; Kim, 2018). Above transition mutations, transversion editing was first achieved using CGBEs and AYBEs, substantially expanding the range of possible nucleotide changes. Prime editing (PE), which combines a Cas9 nickase with reverse transcriptase and pegRNAs, enable all 12 base substitutions as well as precise insertions and replacements. While plant PE efficiencies remain modest, rapid advances in pegRNA design, suppression of mismatch repair, and development of novel viral fusion scaffolds are rapidly closing this efficiency gap. Collectively, base and prime editors offer complementary routes for accurate sequence editing, creating multiple pathways for optimizing traits, and build redundancy into engineered genomes (Yan et al., 2022).

Proteomic technologies have emerged as complementary approaches to genetic tools for evaluating editing outcomes within the context of cellular regulation. Recent advances in cross-linking mass spectrometry, isotopic labeling, and single-cell proteomics facilitate the characterization of transient protein-protein interactions, defining cell type-specific proteomes and connecting genetic modifications to functional signaling networks. For example, studies mapping MAP kinases complexes, prohibits assemblies, and H^+^-ATPase regulatory modules illustrate how proteomics can reveal stress responsive interactions that underlie metabolic resilience (Nguyen et al., 2020). Subsequently, when combined with precise base and prime editing, proteomic mapping transforms genetic interventions into predictive engineering strategies, enhancing confidence in relating genotypic changes to cellular phenotypes (Yan et al., 2022). Additionally, targeted metabolomics provides another layer of assurance by quantifying specific metabolites under defined chromatographic and mass spectrometric conditions. While GC-MS is the preferred method for analyzing primary metabolites due to its compatibility with robust electron ionization libraries and its ability to reproducibly separate compounds, LC-MS/MS platforms extend coverage to include phytohormones, glycolytic intermediates, and secondary metabolites (Yuan et al., 2022). The use of 2-dimensional GC, triple quadrupole MS, and isotopically labeled internal standards enables highly accurate quantitation, while mass spectrometry imaging integrates this quantitative information with spatial maps of metabolites distribution at the tissue and cellular levels. Ultimately, metabolomics integrates concentration data and spatial resolution to provide comprehensive insights into metabolic hot-spots and cross-talk within tissues, which are key characteristics for developing resilient engineered systems (Shen et al., 2023).

## Plant metabolism through the lens of pikobodies, synthetic biology, and single-cell applications

The recognition of plant metabolism as a distributed, condition-dependent information processing system, rather than merely a catalogue of linear pathways e.g., carbon, nitrogen, redox metabolism, among others indicates that specialized metabolite modules dynamically reroute metabolic flux through enzyme assemblies, compartmental interfaces, and regulatory condensates, thereby encoding developmental and stress memory through distinct combinations of biochemical states (Sweetlove et al., 2025). This perspective necessitates a methodological shift. If metabolic phenotypes depend on where reactions occur, when enzymes are activated, and which cell type(s) contribute to flux within a specific microenvironment, then the primary challenge is not merely the identification of metabolic pathway, but the programmable control of molecular states that enables the systematic dissection of metabolic heterogenicity, dynamic changes and targeted interventions. Remarkable advances in three technologies (pikobodies, plant synthetic biology and single-cell/spatial multi-omics) are now converging to enable experimental control, and the examination of engineered metabolism with a degree of spatial and temporal resolution comparable to native cellular granularity (Qin et al., 2025). Pikobodies (intracellular nanobody-like binders), derived from camelid heavy-chain-only antibodies (VHHs), provide an unprecedented capacity to interact with plant metabolism. These molecules function at the protein level within living cells and therefore act on the executive layer of metabolic regulation, including enzyme activity, localisation, stability, and protein-protein interactions, without permanently altering the genome (Jobling et al., 2003; Bakhshandeh, 2023). Unlike conventional overexpression or knockout strategies, which often obscure post-transcriptional regulation and micro-compartmentalized substrate channeling, pikobodies enable subcellular precision through direct modulation of protein conformation or complex assembly within defined cellular compartments. This distinction is important because many critical metabolic traits are regulated by rapid allosteric switching, proteostasis dynamics and metabolon formation rather than solely by transcriptional abundance. In practice, pikobodies can be designed to (i) enable pikobody based immunodetection (ii) confer pikobody-mediated resistance against plant pathogens and relocalize engineered metabolites to prevent self-recognition within their native microenvironments, thereby preventing potential auto-immune responses (iii) protect rate limiting enzymes from ubiquitination mediated degradation and prolonging catalytic half-life, or (iv) disrupt interaction surfaces of proteins involved in the nucleation of multienzyme complexes and metabolons, thereby redistributing flux between competing metabolic branch points (Wang et al., 2021; Bakhshandeh, 2023; Gao and Schornack, 2023; Kumar et al., 2024). Their precision extends beyond conceptual advantage; it enables conditional metabolic perturbations that are inducible, reversible, and cell-type specific, thereby allowing causal inference between enzyme states and resulting metabolite profiles under physiologically relevant conditions. Representative examples showing how this control logic operates across different metabolic pathways are given below. In flavonoid metabolism, enzyme engineering strategies that inhibit Chalcone Synthase (CHS; a member of the CH4 enzyme family) transiently reduce carbon flux into downstream flavonoid branches. This inhibition leads to upstream accumulation of P-Coumaroyl-CoA, increasing its availability for alternative metabolic routes and redirecting flux towards lignin or stilbene biosynthesis (Weng and Chapple, 2010; Kallscheuer et al., 2016). In terpenoid metabolism, targeting 1-deoxy-d-xylulose-5-phosphate reductoisomerase (DXR) in the plastidial MEP pathway can prevent proteasomal degradation, thereby increasing enzymatic stability and extending catalytic half-life, ultimately enhancing terpenoid yield (Li et al., 2013a). In anthocyanins modules – selective interference with the interaction interface between dihydroflavonol 4-reductase (DFR) and anthocyanidin synthase (ANS) alters substrate channeling efficiency and redistributes spatial flux within the pathway (Li et al., 2026). In phenylpropanoid metabolism – sterically blocking of the allosteric site of 4-coumarate coenzyme A ligase (4CL) relieves naringenin-mediated feedback inhibition, thereby maintaining catalytic activity during stressed induced metabolite accumulation (Alberstein et al., 2012). Beyond flux gating, pikobodies can also function as intracellular metabolite sensors. Pikobodies fused to the immune receptor complex Pikm-1-Pikm-2 from the NLR family in rice binds to HMA domains and function as reporter scaffolds to monitor the rice blast fungus *magnaporthe oryzae*, while simultaneously initiating metabolite-associated complex assembly and hormonal-mediated defense pathways (Castel et al., 2024). This dual actuator–sensor capacity positions pikobodies as dynamic rheostats within engineered metabolic circuits, rather than static inhibitors. The transformative step, arises from embedding pikobody-based modulation within synthetic biology architectures. Modern plant synthetic biology is rapidly advancing towards the development of standardized modules governed by the Design–Build–Test–Learn (DBTL) framework, enabling iterative modification of pathway topology and regulatory logic (Vazquez-Vilar et al., 2023). The establishment of standard genetic modules, including quantitative synthetic promoter (TADs), synthetic gene circuits such as ABAleons, ABACUS and SNACKS for monitoring hormone levels (Abscisic acid levels), for jasmonate reporter (jas9-VENUS), riboregulators (npcRNA), modular transcription factors, and programmable logic gates—now permits spatiotemporal tuning of metabolic genes (Laporte et al., 2007).

Assembly platforms such as Golden Gate and MoClo cloning systems facilitate high-throughput circuit construction with enhanced orthogonality and predictability (Chiasson et al., 2019). Within this modular framework, pikobodies function as programmable metabolic actuators, and recent circuit-level innovations further expand their transformative potential. The synthetic developmental regulator module WUSCHEL2 and BABY BOOM (WUS2–BBM) restores embryogenic competence by coordinating meristem identity with somatic cell reprogramming, substantially improving regeneration efficiency in otherwise recalcitrant genotypes (Wang et al., 2025). Integration WUS2–BBM with modular metabolic circuits enables the deployment of engineered pathways into elite cultivars without genotype restriction, thereby bridging laboratory design and field translation. Parallel advances in enzyme engineering complement pikobody-based modulation. PCR-driven saturation mutagenesis of CHS has generated variants with elevated flavonoid output, which can be visually detected through enhanced naringenin chalcone pigmentation (Lyu et al., 2022). Rational metabolic engineering of 4CL and arogenate dehydratase produced feedback-insensitive variants that sustain phenylpropanoid and lignin flux (Beirs et al., 2025). CRISPR-assisted editing of SlGAD2 and SlGAD3 in tomato has produced γ-aminobutyric acid (GABA) shunt variants exhibiting multi-fold increases in fruit GABA accumulation (Nonaka et al., 2017). Replacement of plant thiamine thiazole synthase, a single-turnover suicide enzyme, with catalytic-cycle-sustaining prokaryotic homologs improves thiamine biosynthesis while reducing metabolic cost (Joshi et al., 2020). These examples illustrate the convergence of structural enzymology and programmable control, in which engineered catalytic properties can be integrated with pikobody-mediated post-translational regulation. Single-cell and spatial omics supply the essential readout layer—by generating an atlas of metabolic heterogeneity across tissues and microdomains. Bulk metabolomics often obscures rare but decisive producer cell populations. Single-cell transcriptomics, coupled with spatial mapping, reconstructs cell-state trajectories during development or stress (Yao et al., 2025). However, metabolic output frequently diverges from transcriptional abundance because of compartmentalization, transport constraints, and enzyme complex assembly. Technologies such as matrix assisted laser desorption/ionization – mass spectrometry imaging (MALDI-MSI), desorption electrospray ionization (DESI), and laser ablation electrospray ionization (LAESI) now permit *in situ* visualization of metabolite gradients within intact plant tissues. These platforms reveal subcellular compartmentalization, identify tissue-specific hotspots of defense metabolite accumulation, and resolve inducible microdomains that remain undetected in homogenized extracts (Liu et al., 2025c). When spatial metabolite maps are aligned with single-cell transcriptional atlases, the engineering target shifts from “a pathway” to a cellular circuit topology identifies producer cells, importer cells, and interfacial transport bottlenecks. Integration remains a central bottleneck, because spatial transcriptomics and metabolomics differ in resolution, sensitivity, and noise structure. Consequently, computational frameworks for spatially constrained dimensionality reduction, multimodal data integration, and cross-platform harmonization are essential to minimize engineering artifacts arising from dissociation stress or sampling bias (Tian et al., 2025). Within a DBTL loop, these integrative models identify key control nodes–including enzyme complexes, flux control points, pathway architecture, intracellular transporters, biomolecular condensates and transcriptional regulators—and predict optimal intervention strategies that translate desired biological functions into specific genetic designs within defined cell types (Schwender, 2023; Clark-ElSayed et al., 2025; Jiang et al., 2025; Qin et al., 2025). Taken together, pikobodies engineered enzymes, synthetic circuit modules, and spatial omics reframe plant metabolic bioengineering as a spatio-temporal control problem. Therefore, the new strategy becomes clear: (i) determine metabolic heterogeneity at the single-cell resolution, (ii) quantify control coefficients at the protein and multienzyme complex levels, (iii) implement reversible and conditional interventions through synthetic circuits coupled with pikobody-based actuators, and (iv) validate biochemical outputs within native tissue architecture using spatial metabolomics and computational inference (Sweetlove et al., 2025). Under this comprehensive paradigm, the measurable aspects of plant metabolism become programmable according to their intrinsic architecture, encompassing localization, temporal dynamics, and environmental responsiveness. This alignment provides a durable stress resistance, precise defense deployment, and optimized bioproduction without compromising overall plant growth integrity.

## Artificial engineering in plant metabolism

Beyond conventional nucleases, synthetic biology increasingly adopted artificial engineering strategies that embed resilience and flexibility into metabolic pathways. Transcriptional activators illustrate this trend: early viral effectors such as VP64 offered modest gene induction but limited compatibility with plant chromatin, motivating the development of plant-adapted domains such as EDLL from AP2/ERF transcription factors. Mechanistic refinements such as SunTag and SAM increase activator recruitment density at promoters, while hybrid designs such as VPR combined multiple activation domains for synergistic upregulation (Liu et al., 2023a, Liu et al., 2025a). Platforms such as CRISPR-Act3.0 and dCasEV2.1 build on these strategies by enabling coordinated activation of multiple biosynthetic genes, effectively redirecting entire metabolic fluxes (Pan et al., 2021). dCas-based fusion systems broaden regulatory capabilities beyond gene activation to include repression, epigenetic modification, and higher-order chromatin remodeling. By tethering histone acetyltransferases, demethylases, or DNA methyltransferases, dCas effectors can rewrite chromatin landscapes, establishing heritable transcriptional states (Laforest and Nadakuduti, 2022). Aptamer-based multiplexing further enables simultaneous activation and repression across gene networks, creating programmable transcriptional plasticity that underpins bet-hedging strategies in plant metabolism.

AI provides an additional layer of programmable control. ML and deep learning platforms are increasingly applied to plant metabolism to accelerate cell-type characterization and pathway modeling. Tools such as Biological Image Analysis Software (BIAS) and DIABLO integrate histological imaging with single-cell omics to define cell identities, predict metabolic states, and map pathway activities (Singh et al., 2019). Extending these frameworks to plants, allows metabolic engineers to target specific cell populations, quantify biomass allocation, and predict flux outcomes before intervention. Interaction-predictive models such as MESSI further enhance precision by inferring intercellular signaling from ligand–receptor networks, offering strategies to reveal metabolic crosstalk across tissues (Sears et al., 2024). While plant-specific dataset remain limited, emerging resources such as the plant cell marker database and PlantPhoneDB, combined with AI-driven image augmentation, are progressively overcoming these barriers (Xu et al., 2022a). Collectively, the integration of CRISPR-based transcriptional regulation and AI-driven predictive modeling establishes a robust framework for the artificial engineering of plant metabolism, embedding adaptability and predictive control into synthetic designs. Synthetic epigenetics represents an emerging frontier in artificial engineering. Locus-specific chromatin modification using dCas-DNMT fusions, TET1 demethylase, or histone-modifying effectors enables precise and reversible tuning of gene expression without altering underlying DNA sequences (Pulecio et al., 2017). RNA-level engineering is increasingly integrated into this framework, with synthetic m6A “read–write” circuits capable of propagating epi-transcriptomic states through engineered initiator and effector modules (Park et al., 2019). Deep learning models now accurately predict DNA methylation, histone modifications, and RNA marks, enabling rational epigenetic circuit design.

This integration of predictive AI with programmable epigenetic editing platforms offers a strategic safeguard against the inherent inflexibility associated with permanent DNA edits, enabling reversible and dynamic modulation of phenotypes in response to changing environmental conditions (Zhang et al., 2025c). These integrated frameworks have broader applicability in the advancement of SMART crops and plant factory systems. SMART crops integrate biosensors with engineered genetic circuits to detect drought stress, pathogen exposure, and temperature extremes converting these environmental cues into adaptive morphological and metabolic responses, including, enhanced culm mechanical strength and reprogrammed leaf physiology. In parallel, plant factory systems employ synthetic gene circuits to transform crops into industrial-scale bioreactors producing antibodies, vaccines, and nutraceuticals compounds (Wang et al., 2024). Incorporating, AI-guided circuit design, biosensor-based regulatory control, and programmable epigenetics systems, increasingly positions plants as programmable chassis for both adaptive agriculture and high-value biomolecule production.

Genetic circuit optimization increasingly relies on multi-omics frameworks that integrate DNA replication, transcriptional, and translation regulation, and higher-order chromatin organization into predictive DBTL workflows (Endo et al., 2019). Technological advances in nanopore sequencing, bisulfite sequence-based DNA methylation mapping, ATAC-seq, and Hi-C technologies provide high-resolution characterization of genome architecture and epigenetic modifications, enabling circuit designs that accounts for chromatin accessibility and 3-dimensional genome folding. Genomic selection accelerates trait improvement by calculating genomic estimated breeding values (GEBVs) that integrate genome-wide marker data with phenotypic and environmental variables, to generate “physiologically-informed GEBV” that accurately predict yield performance under stress conditions (Krishnappa et al., 2021). AI further enhances predictive accuracy by embedding multi-omics datasets into ML models to optimize trait prioritization and genetic module selection. AI-supported, target-oriented prioritization strategies integrate molecular and whole-plant traits to improve predictions of breeding outcomes and facilitate the identification of superior genotypes (Zhang et al., 2025a). At the molecular level, AI-driven protein engineering using models such as AlphaFold and Protein-MPNN accelerates the rational design of novel enzymes and improves the stability of heterologous protein expression in plant systems. The computational platforms of these tools complement experimental directed evolution approaches, by enabling high-throughput *in silico* screening and iterative optimization of synthetic biocatalysts (Cadet et al., 2022). Large language models further extend optimization across breeding pipelines. CRISPR-GPT automates sgRNA design, delivery method selection, and validation workflow planning, while BioGPT, BiomedGPT, and CropGPT integrate multi-omics, climate, and agronomic datasets into iterative breeding workflows (Zhu et al., 2024). Establishing continuous learning system, reduces experimental iteration and strengthens predictive control, thereby accelerating synthetic circuit optimization. Optimization extends to architectural traits and metabolic flux reprogramming. multiplex genome editing of plants male sterility genes including MS26, MS45 in maize and prime editing in rice prone to genetic improvements and generates novel hybrid lines, while targeted modification of plant height and branching regulators reshapes plant architecture to enhanced yield performance (Svitashev et al., 2016; Li et al., 2025). Directed evolution of P450 enzymes, miRNA circuit rewiring (e.g., osa-miR156, zma-miR164), and multiplex CRISPR-based editing of yield-related loci exemplify strategies for fine-tuning modulation of developmental and metabolic traits (Chen et al., 2015). Integrating these molecular, cellular, and whole-plant interventions, shifts metabolic bioengineering transitions from incremental modifications toward predictive and system-level circuit optimization.

The variety of metabolic engineering technologies that can improve plant defenses has been impressive. However, each is at a different stage of development and not yet ready for translation into practice. Genome editing using the CRISPR/Cas system is the most developed. This is because it offers an extraordinary degree of precision, scalability, and success in deploying these tools in multiple crop improvement initiatives. A recent series of improvements in genomic selection, marker-assisted breeding, and predictive breeding frameworks also augment genome editing applications. These advances move toward the development of climate-resilient crops and accelerate trait deployment (Mohd Saad et al., 2022). Inducible promoters and synthetic transcriptional circuits provide the greatest increase in spatio-temporal control over plant defense-related signaling pathways. However, they are still mostly used in controlled experimental systems. The reason is that many technical problems—such as instability, complex regulation, and environmental sensitivity—still need to be solved before broad adoption. Volatile-mediated engineering and microbial community-informed metabolic engineering are sustainable alternatives. These enhance plant resilience through ecologically based interactions. While these alternative methods show promise in providing new mechanisms for plant resilience, there are major concerns about variability in responses across environments. Large-scale field testing is needed to validate efficacy.

Single-cell omics, spatial metabolomics, and fluxomics have emerged as exciting platforms for discovery. They help identify key regulatory points or metabolically limiting steps in plants. While all three types of analysis have outstanding capabilities for discovering new knowledge about plant metabolism, they are limited by high cost, technical complexity, and limited access. Metabolic modeling using artificial intelligence and machine learning algorithms offers enormous opportunities for designing and optimizing pathways. Unfortunately, these prediction opportunities are heavily dependent on the availability of large quantities of accurate biological data. RNAi-enabled targeted pest suppression and precision crop protection applications, when combined with nanotechnology, have yielded promising preliminary results. However, progressing to large-scale commercial deployment, biosafety evaluations, and regulatory approvals will require ongoing intensive research (Yu et al., 2023b).

## Outlooks & future directions

**Figure 5:**
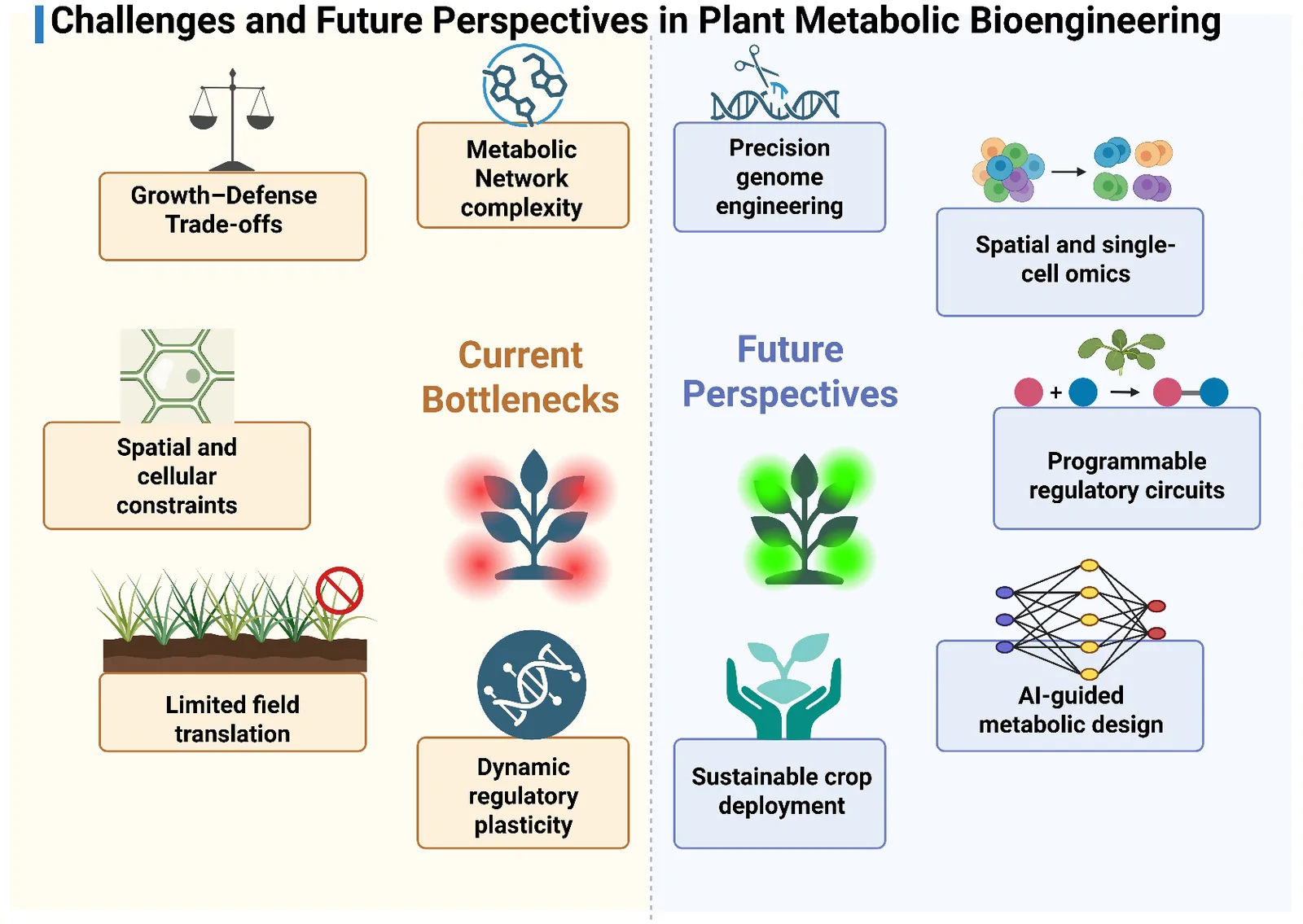
Challenges and future perspectives in plant metabolic bioengineering. The schematic summarizes the major biological, technical, and translational challenges limiting metabolic bioengineering, including metabolic network complexity, spatial compartmentalization, regulatory plasticity, and growth–defense trade-offs. It also highlights emerging strategies, including precision genome editing, spatial multi-omics, synthetic regulatory circuits, and AI-assisted modeling, that facilitate programmable, cell-specific, and environmentally responsive regulation of defense-associated metabolic pathways for sustainable crop improvement.

The rapid convergence of high-resolution multi-omics, advanced genome editing, and predictive computational modeling is redefining both the scope and goal of plant metabolic bioengineering (PME). Over the past two decades, multiple translational proof-of-concept studies have shown that targeted modulation of plant metabolic pathways via DBTL cycles enhances the accumulation of high-value specialized metabolites in crops and medicinal plants. Despite significant advances in transient pathway expression and synthetic promoter engineering, the stable reconstruction of complex metabolic circuits, particularly those controlling plant defense, remains elusive. This gap is attributed to the limited number of transformations attempts and persistent challenges in multi-gene vector assembly, the restricted availability of tightly regulated promoter systems, and phytotoxicity caused by the overaccumulation of reactive intermediates and defense-related metabolites. Addressing these limitations requires next-generation PME to priorities the development of modular, cell-type–specific, and stimulus-inducible expression cassettes and moderate metabolite production to target tissues, such as glandular trichomes, laticifers, or vascular parenchyma. Concurrent implementation of compartmentalized design within metabolically insulated cellular domains will minimize interference with growth-essential pathways. However, the enzymatic promiscuity and functional redundancy among endogenous multi-substrate enzymes remain key barriers to pathway specificity, often diverting precursor flux or triggering feedback inhibition. Genome-wide CRISPR-based screening approaches, combined with metabolite-responsive biosensors, offer a systematic strategy to identify and counteract these bottlenecks; however, these approaches require parallel advances in spatial metabolite mapping and comprehensive transporter annotation. Future progress requires detailed characterization of subcellular metabolite landscapes—through spatial metabolomics, fluxomics, and high-resolution localization of membrane transporters to accurately target and insulate biosynthetic fluxes. Plastid genome engineering represents a powerful but underutilized strategy for redirecting metabolic flux; however, its effective implementation depends on coordinated optimization of transport dynamics and enzyme localization within predictive modeling frameworks. Integrating constraint-based metabolic models (e.g., FBA, ME, and ETFL-models) with real-time transcriptomic and proteomic data enables in silico simulations of defense–growth trade-offs under complex environmental conditions. In parallel, ML platforms such as DLKcat and other deep enzyme-function prediction systems are revolutionizing enzyme engineering by predicting flux bottlenecks, substrate specificity, and reaction kinetics—thereby accelerating the *de novo* construction of biosynthetic pathways. AI-guided genetic circuit design emerges as a foundational strategy for pyramiding defense pathways, particularly when combined with dynamic, feedback-regulated modules that modulate metabolite outputs in response to pathogen pressure or environmental signals. Nevertheless, the efficient deployment of these computationally enabled frameworks requires the establishment of standardized data repositories, curated metabolic libraries, and plant-specific design platforms broadly accessible to the research communities. The limited representation of plant chassis in synthetic biology platforms requires correction through coordinated integration of experimental biology, bioinformatics, and system-level design. Looking ahead, PME is poised as both a method for metabolic enhancement and a framework for anticipatory immunity and scalable biosynthesis in crops. Extending synthetic design to epigenetic and post-transcriptional regulatory layers, such as long noncoding RNAs, chromatin remodelers, and microRNA networks, enables engineered defense circuits to achieve precise temporal and spatial control, thereby minimising metabolic burden while maximizing resilience. Furthermore, recent advances in understanding microbiome–host metabolite crosstalk create opportunities for designing microbial consortia that function synergistically with engineered pathways. Overall, future progression depends on developing predictive, modular, and adaptive metabolic systems capable of responding to real-time endogenous and environmental signals. The evolution of PME from an empirical discipline to a model-driven, AI-enabled engineering paradigm will be crucial for addressing global challenges, including crop protection, climate resilience, and the development of sustainable bio-based economies. This review highlights several unresolved but pressing questions (see **Box 1**; **Figure 5**) that define the conceptual, technical, and translational frontiers of plant metabolic defense engineering, and outlines priorities for forthcoming research.

Box 1 **Open questions in metabolic bioengineering for plant defense**

- How can engineered defense circuits be selectively insulated from central metabolic flux to minimize deleterious trade-offs with developmental, reproductive, or stress-adaptive pathways in planta?
- What mechanistic principles and spatial constraints determine the optimal subcellular compartmentalization and temporal deployment of volatile and non-volatile defense metabolites?
- Can metabolite-responsive biosensors and autonomous feedback-regulated modules be integrated into plant systems to dynamically detect biotic stress and reallocate defense metabolites in real time?
- How do plant-associated microbiota modulate or disrupt synthetic metabolic pathways, and can microbial consortia be rationally designed to function in synchrony with host-engineered immunity?
- Which artificial intelligence architectures most accurately predict enzymatic cross-reactivity, metabolic constraints, and emergent feedback regulation within high-dimensional engineered defense networks?

Beyond pathway construction and metabolic flux optimization, a largely underexplored frontier in PME is the evolutionary robustness and long-term stability of engineered metabolic traits under variable field conditions. The future of plant metabolic bioengineering will move away from current static pathway modification toward the development of Programmable, Adaptive, and Predictive Biological Systems. Technologies beyond Genome Editing that have emerged include CRISPR Activation/Inhibition (CRISPRa/CRISPRi), Prime Editing and Base Editing, which collectively offer new levels of precision to dynamically regulate Defense-Associated Metabolism Pathways via minimal or no Genomic Disruption. Advances in Spatial Metabolomics, Single-Cell Transcriptomics, and High-Resolution Imaging Mass Spectrometry are also enabling visualization at the cellular level of metabolite distribution and immune response. Additionally, the integration of artificial intelligence (AI), machine learning (ML), and digital twin metabolic models may enable data-driven prediction of pathway bottlenecks, optimizing metabolic flux-based modeling, and the identification of candidate engineering targets. While each technology has different levels of Maturity, Scalability, and Readiness for Translational Use, comparing these technologies provides significant insight into future Crop Improvement Strategies. A comparative overview of major technologies currently shaping the field is presented in **Supplementary Table 3**.

Despite the remarkable advances in metabolic bioengineering, several challenges continue to limit the translation of laboratory-scale innovations into commercially viable crop improvement strategies. The performance of engineered metabolic pathways is often influenced by genotype-specific responses, environmental variability, metabolic burden, and unintended effects on plant growth, fitness, and ecological interactions. Furthermore, the long-term stability of engineered traits under diverse field conditions remains insufficiently validated. Regulatory approval processes for genetically engineered and genome-edited crops vary considerably among countries, creating additional barriers to technology deployment and commercialization. Biosafety considerations, including off-target effects, unintended metabolic alterations, impacts on non-target organisms, and ecosystem-level consequences, require rigorous assessment before large-scale adoption. Equally important is public perception and societal acceptance, which continue to influence policy decisions and market uptake of engineered crops. Therefore, future research should integrate systems-level risk assessment, long-term field validation, transparent regulatory frameworks, and stakeholder engagement to ensure that advances in metabolic bioengineering can be translated into sustainable, safe, and socially acceptable agricultural solutions.

Most current engineering efforts are evaluated under controlled environments; even so, defense metabolism functions within dynamic ecological contexts shaped by temperature variability, nutrient limitation, pathogen diversity, and microbiome turnover. Therefore, future PME frameworks must incorporate evolutionary and ecological modeling to anticipate the response of engineered circuits to selective pressures across seasons and generations. Advances should prioritize the transition from controlled-environment demonstrations to field-validated applications by integrating ecological risk assessment, regulatory considerations, and socioeconomic acceptance into metabolic bioengineering pipelines. Consequently, a clear distinction must be made between proof-of-concept technologies that demonstrate biological feasibility and those approaching translational readiness for agricultural implementation. In this context, the translational readiness categories summarized in **Supplementary Table 3** provide a qualitative framework for interpreting the current developmental status of representative metabolic bioengineering technologies. Technologies categorized as field-ready have demonstrated successful implementation or advanced validation in crop improvement or field-relevant environments, whereas mature technologies are supported by substantial experimental evidence across multiple plant systems. Emerging technologies have shown promising performance primarily under controlled laboratory or greenhouse conditions, while conceptual or early-stage approaches remain largely supported by computational predictions or proof-of-concept studies and therefore require further biological and field validation before practical deployment. These classifications are intended to provide readers with an evidence-based perspective on the maturity, applicability, and future development of each engineering strategy rather than representing fixed categories, acknowledging that translational readiness will continue to evolve as new experimental evidence becomes available. Future success in plant metabolic bioengineering will depend not only on technological innovation but also on the integration of field validation, regulatory compliance, ecological sustainability, and stakeholder acceptance to ensure responsible and effective deployment of engineered crop systems. A closely related priority is to understand metabolic memory and defense priming durability, including whether engineered epigenetic states and inducible defense programs are stably inherited or require periodic reactivation. Another emerging direction emphasizes metabolite deployment rather than simple accumulation, focusing on engineering biosynthetic capacity and controlled release, conjugation, volatilization, or sequestration to maximize defensive efficacy while minimizing intracellular toxicity. Advances in cell-to-cell transport engineering, vesicle trafficking control, and metabolite export systems may redefine the spatial distribution of defense compounds within tissues and their temporal dynamics during infection. Finally, successful agricultural translation will require integrating PME with breeding pipelines, regulatory frameworks, and life-cycle assessments to ensure that engineered metabolic traits are not only biologically effective but also agronomically viable, environmentally sustainable, and socioeconomically acceptable. Addressing these factors will elevate PME from a molecular optimization strategy to a systems-level design framework capable of delivering durable, adaptive, and context-responsive plant immunity.

In conclusion, axiomatic and modular approaches in PME open new avenues for deciphering gene regulatory circuits, allowing scientists to enable more rapid plant designs expeditiously and provide strategic solutions to overcome the grand challenges of our time, such as, plant resilience, to climate and environmental changes.

## Glossary

ABA: abscisic acid
AI: artificial intelligence
ANS: anthocyanidin synthase
AP2/ERF: APETALA2/ethylene-responsive factor
ATAC-seq: assay for transposase-accessible chromatin with sequencing
bHLH: basic helix-loop-helix
BGC: biosynthetic gene cluster
BXs: benzoxazinoids
CDPKs: calcium-dependent protein kinases
CHS: chalcone synthase
CMV: cucumber mosaic virus
COI1: coronatine insensitive 1
DBTL: Design–Build–Test–Learn
DFR: dihydroflavonol 4-reductase
DMAPP: dimethylallyl pyrophosphate
DRPs: defense-related proteins
dCas9: dead Cas9 (catalytically inactive Cas9)
E4P: erythrose-4-phosphate
ET: ethylene
ETFL: enzyme-constrained thermodynamic flux models
ETI: effector-triggered immunity
FPP: farnesyl pyrophosphate
GABA: γ-aminobutyric acid
GC-MS: gas chromatography-mass spectrometry
GEMs: genome-scale metabolic models
GGPP: geranylgeranyl pyrophosphate
GLVs: green leaf volatiles
GPP: geranyl pyrophosphate
H3K4me3: histone H3 lysine 4 trimethylation
HDA6/HDA9: histone deacetylase 6/9
IPP: isopentenyl pyrophosphate
JA: jasmonic acid
LAESI: laser ablation electrospray ionization
LC-MS/MS: liquid chromatography-tandem mass spectrometry
LOX: lipoxygenase
MALDI-MSI: matrix-assisted laser desorption/ionization mass spectrometry imaging
MAPKs: mitogen-activated protein kinases
ME: metabolic engineering
MEP: methylerythritol phosphate
METS1: methionine synthase
ML: machine learning
MVA: mevalonate
NADPH: nicotinamide adenine dinucleotide phosphate (reduced)
NHP: N-hydroxypipecolic acid
NLR: nucleotide-binding leucine-rich repeat
NMR: nuclear magnetic resonance
NPR1: non-expressor of pathogenesis-related genes 1
OPDA: 12-oxo-phytodienoic acid
ORCA3: octadecanoid-responsive Catharanthus AP2/ERF domain 3
PAL: phenylalanine ammonia-lyase
PAM: protospacer adjacent motif
PAMP: pathogen-associated molecular pattern
PE: prime editing
PEP: phosphoenolpyruvate
PICI1: Pik-1-interacting component 1
Pikm: Pik major resistance gene
PPP: pentose phosphate pathway
PR: pathogenesis-related
PRRs: pattern recognition receptors
PVY: potato virus Y
QTL: quantitative trait locus
ROS: reactive oxygen species
SA: salicylic acid
SAM: S-adenosylmethionine
SAR: systemic acquired resistance
SCMV: Sugarcane mosaic virus
sgRNA: single guide RNA
SNACKS: synthetic sensor for abscisic acid
TFs: transcription factors
TMV: tobacco mosaic virus
TPS: terpene synthase
UHPLC-HRMS: ultra-high performance liquid chromatography-high resolution mass spectrometry
VHH: variable domain of heavy-chain-only antibody
VOC: volatile organic compound
WRKY: WRKY transcription factor family
WUS2–BBM: WUSCHEL2–BABY BOOM
