# Supplementary material for: A voyage of reprogrammable metabolic bioengineering reshapes plant defense: from editing tools to synthetic systems
Source: Front Plant Sci. 2026 Aug 26;17:1842722. doi: 10.3389/fpls.2026.1842722 (PMC13555926; doi:10.3389/fpls.2026.1842722)
Supplement: Supplementary file 1 [file DataSheet1.pdf]

Supplementary table 1. Major classes of defense-associated metabolites involved in plant immunity and their metabolic bioengineering potential. The table summarizes representative defense metabolite classes, their biosynthetic origins, major immune functions, biotic targets, and potential engineering strategies for enhancing plant resistance. Information was synthesized and adapted from studies on secondary metabolites, plant immunity, metabolic pathway regulation, and defense-associated biosynthetic networks, together with information compiled and interpreted from the present review.

Metabolite Class	Representative Compounds	Biosynthetic Origin	Major Immune Functions	Major Biotic Targets	Potential Bioengineering Strategies
**Phenylpropanoids**	Lignin, coumarins, phenolic acids, flavonoid precursors	Phenylalanine pathway	Cell wall reinforcement, antioxidant defense, phytoalexin production	Fungi, bacteria, wound-associated pathogens	PAL, C4H, 4CL overexpression; MYB/WRKY engineering; flux optimization
**Flavonoids**	Anthocyanins, flavanols, flavones, isoflavonoids	Phenylpropanoid pathway	ROS scavenging, signaling, antimicrobial activity	Fungi, bacteria, herbivores	CHS/CHI engineering; transcription factor regulation; pathway stacking
**Terpenoids**	Monoterpenes, sesquiterpenes, diterpenes, phytoalexins	MVA and MEP pathways	Direct toxicity, volatile-mediated defense, predator recruitment	Herbivores, fungal pathogens	TPS engineering; compartmentalized biosynthesis; synthetic metabolons
**Alkaloids**	Nicotine, camalexin, benzylisoquinoline alkaloids	Amino acid-derived pathways	Chemical defense and antimicrobial activity	Insects, fungi, bacterial pathogens	Pathway reconstruction; CRISPR-guided metabolic redesign
**Glucosinolates**	Indolic and aliphatic glucosinolates	Amino acid-derived metabolism	Defense activation via hydrolysis products	Herbivores, pathogens	MYB28/MYB29 manipulation; glucosinolate pathway optimization
**Benzoxazinoids**	DIMBOA, DIBOA, MBOA	Indole-derived metabolism	Root defense, rhizosphere protection, antimicrobial activity	Root pathogens, herbivores	BX pathway engineering; microbiome-guided metabolic enhancement
**Phytoalexins**	Camalexin, resveratrol, sakuranetin	Multiple specialized pathways	Inducible antimicrobial defense	Fungi, bacteria	Inducible promoters; pathogen-responsive engineering
**Volatile Organic Compounds (VOCs)**	Green leaf volatiles, terpenoid VOCs, methyl salicylate	Fatty acid and terpenoid metabolism	Defense signaling, priming, tritrophic interactions	Herbivores and pathogen-associated stress	VOC pathway engineering; synthetic regulatory circuits

Supplementary table 2. Evidence-based assessment of representative metabolic bioengineering strategies for improving plant immunity.

Strategy	Representative application	Experimental evidence	Translational readiness	Major limitation	Representative crop/pathosystem
**CRISPR/Cas genome editing**	Editing susceptibility or defense genes	Multiple validated studies	High	Regulatory variation; off-target assessment	Rice, Tomato
**CRISPRa/i and synthetic TFs**	Programmable activation of metabolic pathways	Proof-of-concept	Medium	Delivery efficiency; regulatory complexity	Arabidopsis, Nicotiana
**Transcription factor engineering**	MYB, WRKY, ERF regulation of specialized metabolism	Well validated	Medium-High	Growth–defense trade-offs	Grapevine, Catharanthus
**Pathway compartmentalization**	Organelle and tissue-specific metabolite production	Experimental	Medium	Engineering complexity	Tobacco, Arabidopsis
**Spatial metabolomics**	Tissue-resolved metabolite mapping	Emerging	Medium	High instrumentation cost	Model plants
**Fluxomics**	Quantification of metabolic flux redistribution	Experimental	Medium	Limited temporal resolution	Arabidopsis
**AI/ML-assisted pathway prediction**	Candidate gene prioritization and pathway optimization	Emerging	Low–Medium	Requires extensive datasets	Multiple crops
**Genome-scale metabolic models (GEMs)**	Prediction of pathway bottlenecks	Proof-of-concept	Medium	Model accuracy depends on annotation quality	Arabidopsis
**Microbiome engineering**	Manipulation of rhizosphere metabolism	Experimental -field trials	Medium	Environmental variability	Wheat, Maize
**Synthetic gene circuits and biosensors**	Inducible defense activation	Early proof of concept	Low	Limited field validation	Nicotiana

Supplementary table 3. Emerging technologies enabling next-generation plant metabolic bioengineering. The table compares major technologies currently applied in plant metabolic bioengineering, including genome editing, synthetic regulatory systems, multi-omics platforms, artificial intelligence-assisted pathway design, and microbiome engineering. Technologies are evaluated according to their primary applications, advantages, limitations, and translational readiness.

Technology	Primary Application	Major Advantages	Major Limitations	Translational Readiness
**CRISPR/Cas Genome Editing**	Targeted pathway modification	High precision; multiplex editing; stable inheritance	Regulatory restrictions; potential off-target effects	High
**Base Editing**	Single-nucleotide modification	No double-strand breaks; precise editing	Editing window constraints	High–Emerging
**Prime Editing**	Precise insertions, deletions, and substitutions	Flexible genome engineering	Lower editing efficiency in plants	Emerging
**Synthetic Promoters**	Conditional pathway activation	Tissue-specific and inducible control	Promoter optimization required	Emerging
**dCas9-Based Regulatory Circuits (CRISPRa/CRISPRi)**	Programmable gene activation/repression	Reversible and multiplex regulation	Complex circuit design	Emerging
**Synthetic Gene Circuits**	Dynamic metabolic control	Programmable and environment-responsive regulation	Limited field validation	Experimental–Emerging
**Fluxomics**	Identification of metabolic bottlenecks	Quantitative pathway optimization	Data-intensive and technically demanding	Emerging
**Spatial Metabolomics**	Tissue- and cell-specific metabolite mapping	High spatial resolution of defense responses	High instrumentation cost	Emerging
**Single-Cell Multi-Omics**	Cellular heterogeneity analysis	Resolves cell-specific metabolic regulation	Complex computational analysis	Emerging
**AI/ML-Assisted Metabolic Design**	Target prioritization and pathway prediction	Accelerates pathway optimization and predictive design	Requires extensive datasets	Emerging–High
**Genome-Scale Metabolic Models (GEMs)**	In silico metabolic simulation	Predictive hypothesis generation	Model-dependent accuracy	Emerging
**Microbiome Engineering**	Rhizosphere and endophyte manipulation	Sustainable ecosystem-level resilience	Environmental variability	Emerging
**RNAi–Nanoparticle Platforms**	Targeted pest and pathogen suppression	Species-specific control; reduced pesticide use	Regulatory and delivery challenges	Emerging
**Biosensor-Guided Metabolic Regulation**	Real-time monitoring of metabolic fluxes	Dynamic pathway control	Limited plant deployment	Experimental
